# Suppressor of rid1 (SID1) shares common targets with RID1 on florigen genes to initiate floral transition in rice

**DOI:** 10.1371/journal.pgen.1006642

**Published:** 2017-02-24

**Authors:** Li Deng, Lingmei Li, Shuo Zhang, Jianqiang Shen, Shaobo Li, Sifan Hu, Qiang Peng, Jinghua Xiao, Changyin Wu

**Affiliations:** National Key Laboratory of Crop Genetic Improvement and National Center of Plant Gene Research (Wuhan), Huazhong Agricultural University, Wuhan, China; Peking University, CHINA

## Abstract

The transition from vegetative to reproductive growth is a critical process in the life cycle of higher plants. Previously, we cloned *Rice Indeterminate 1* (*RID1*), which acts as the master switch for the transition from the vegetative to reproductive phase in rice. Although the photoperiod pathway of *RID1* inducing expression of the florigen genes *Hd3a* and *RFT1* via *Ehd1* has been established, the alternative pathways for the essential flowering transition need to be further examined. Here, we identified a *Suppressor* of *rid1* (*SID1*), which rescues the never-flowering phenotype of *rid1*. *SID1* encodes an INDETERMINATE DOMAIN (IDD) transcription factor. Mutation in *SID1* showed the delayed flowering phenotype. Gain-of-function of *SID1*, *OsIDD1*, or *OsIDD6* could restore the *rid1* to flowering. Further analyses showed SID1 and RID1 directly target the promoter regions of *Hd3a* and *RFT1*, two florigen genes in rice. Taken together, our results reveal an autonomous flowering pathway might be mediated by *RID1*, thereby controlling the phase transition from vegetative to reproductive development in rice.

## Author summary

Transition from vegetative to reproductive phase is a critical developmental switch in the life cycle of higher plants. In rice, our previous work suggested *Rice Indeterminate 1* (*RID1*) acts as the master switch for the transition to flowering. Mutation in *RID1* results in a never-flowering phenotype. In order to uncover the molecular network regulated by *RID1*, a *Suppressor* of *rid1* (*SID1*) was identified in this study. Both *SID1* and *RID1* encode a plant-specific INDETERMINATE DOMAIN (IDD) transcription factor. Overexpression of *SID1*, *OsIDD1*, or *OsIDD6* could rescue the never-flowering phenotype of *rid1*. Molecular data indicate both SID1 and RID1 physically bind the promoters of the florigen genes *Hd3a* and *RFT1* in rice. Thus, we propose that the transition to flowering could be regulated by *RID1* through the autonomous pathway, in addition to the photoperiod pathway.

## Introduction

The post-embryonic development of flowering plants can be divided into two major phases: the vegetative and reproductive growth stages. During vegetative development, shoot apical meristems continue to produce leaves for the generation of organic materials through photosynthesis. After a given number of leaves are generated, endogenous genetic factors and environmental signals control the time of flowering [1]. Molecular regulatory networks that monitor the changes in the environment and complex endogenous signals determine the timing of the developmental transition [2–4]. Great progress has been made in elucidating the molecular basis for the flowering transition in *Arabidopsis*, which represents a long-day (LD) plant [5–10]. Numerous genes were identified and integrated into six major pathways: the photoperiod, vernalization, age, autonomous, gibberellin, and ambient temperature pathways [11]. Rice is not only a leading cereal crop in the world, but also a representative short-day (SD) plant for flowering time (heading date) studies. As an important agronomic trait, heading date is crucial for determining the regional adaptability and grain yields [12–14].

The molecular mechanisms for flowering time control have been well studied in *Arabidopsis*. However, studies of heading date control in rice have almost exclusively focused on the photoperiodic pathway [15]. Although rice is regarded as a SD plant, it also has evolved its flowering pathway to induce flowering under LD conditions during artificial domestication at high latitudes [16–20]. Thus, photoperiodic flowering in rice can be artificially considered as two distinct pathways: the evolutionarily conserved *OsGI-Hd1-Hd3a* pathway for adaption under SD conditions, which is parallel to the *GI-CO-FT* module in *Arabidopsis* [21, 22], and the uniquely evolved *Ghd7-Ehd1-Hd3a/RFT1* pathway for adaptation under LD conditions [12, 13, 15, 23]. To understand the photoperiodic control of flowering in rice more comprehensively, recent investigations have identified some flowering mutants that are insensitive to photoperiod variations. Mutants with *RID1*/*OsID1*/*Ehd2*, a rice ortholog of the maize *INDETERMINATE1* (*ID1*) gene, showed a late- or never-flowering phenotype under SD or LD conditions [24–26], indicating that *RID1* might function as an autonomous factor to induce the floral transition in rice [26]. *Ehd3* encodes a plant homeodomain finger-containing protein [27]. Mutation in *Ehd3* results in no flowering under LD conditions, suggesting that *Ehd3* acts as a flowering inducer in the unique genetic pathway *Ehd3-Ghd7-Ehd1* in rice [27]. In addition, *Ehd4*, encoding a novel CCCH-type zinc finger protein, was identified as a critical regulator promoting flowering under both SD and LD conditions [16]. *ehd4* also showed a never-flowering phenotype under LD conditions [16]. All these flowering switches (*RID1*, *Ehd3*, *Ehd4*) thus far identified in rice and have no direct homologs in *Arabidopsis* [16, 26, 27]. Thus, it appears that *RID1*, *Ehd3*, and *Ehd4* may participate in a rice-specific flowering transition pathway, the underlying molecular mechanisms of which are still not well understood.

*RID1*/*OsID1*/*Ehd2* encodes a highly conserved zinc finger protein in plants [24–26]. The zinc fingers and its surrounding sequence compose a so-called INDETERMINATE DOMAIN (IDD), which was identified in all higher plant genomes [28]. Maize ID1 is the founding member of the IDD family and controls the transition to flowering in maize [29]. *In vitro* DNA binding experiments showed that ID1 binds selectively to an 11-bp DNA sequence with the consensus motif TTTGTCG/CT/CT/aT/aT via the IDD [30]. Sixteen and fifteen IDD members were identified in the genomes of *Arabidopsis* and rice, respectively [28]. Previous studies of IDD members in *Arabidopsis* revealed that *IDD* genes participate in multiple developmental processes. *AtIDD8* is involved in photoperiodic flowering by modulating sugar transport and metabolism [31]. AtIDD8, AtIDD3, and AtIDD10, either physically or genetically interact with the GRAS domain proteins SHR and SCR to regulate root development or patterning [32–34]. *AtIDD1* is required for seed maturation and germination [35]. *AtIDD14*, *AtIDD15*, and *AtIDD16* play a critical role in lateral organ morphogenesis and gravitropism by regulating spatial auxin accumulation [36]. Recent investigations showed that some IDD members (AtIDD2, AtIDD3, AtIDD4, AtIDD5, AtIDD9, and AtIDD10) interact with DELLAs to control gibberellin homeostasis and signaling and modulate flowering time in *Arabidopsis* [37, 38]. *RID1* is the only IDD member being functionally analyzed in rice. *RID1* and its putative orthologs, *ID1* in maize and *SbID* in *Sorghum*, are preferentially expressed in immature leaves and may exhibit conserved function for flowering transition [26, 28, 29].

In maize, *ID1* is a key regulator of the transition from vegetative to reproductive growth [29, 39]. The *id1* mutant has prolonged vegetative growth and retains vegetative features in the inflorescence [29]. The *ID1* gene was proposed to regulate the production or transmission of a mobile florigenic signal [29, 40]. Transcript and metabolite profiles indicated that expression levels of major sucrose and starch metabolism genes were altered in the *id1* mutant, suggesting that *ID1* might be involved in the starch to sucrose transition and sucrose utilization within the leaf [39]. However, similar changes in transitory starch and sucrose are not observed in the photoperiodic flowering plants [39]. Thus, it appears that *ID1* is likely engaged in a novel autonomous flowering pathway that is distinct from the photoperiod induction pathway [39, 41]. Our previous study showed that *RID1* acts as a master switch of the flowering transition in rice [26]. Loss of function of *RID1* seriously suppressed the expression of *Ehd1* and florigen genes *Hd3a* and *RFT1*, suggesting that *RID1* plays important roles in photoperiodic flowering promotion in rice [26]. At present, the direct target of *RID1* and whether *RID1* controls an autonomous flowering pathway in rice remain unclear.

In this study, a gain-of-function mutant *suppressor of rid1-D* (*sid1-D*) was identified. *sid1-D* restored the *rid1* mutant to flowering successfully. SID1 belongs to the IDD family in rice. Loss-of-function mutants of *SID1* exhibit late flowering under LD conditions. Moreover, our results show that RID1 and SID1 directly regulate the expression of *Hd3a* and *RFT1*, two florigen genes in rice. Our results indicate that the original function of RID1 might trigger the expression of florigen genes, thus controlling the flowering transition in rice.

## Results

### Identification of a suppressor of *rid1*

**Fig 1:**
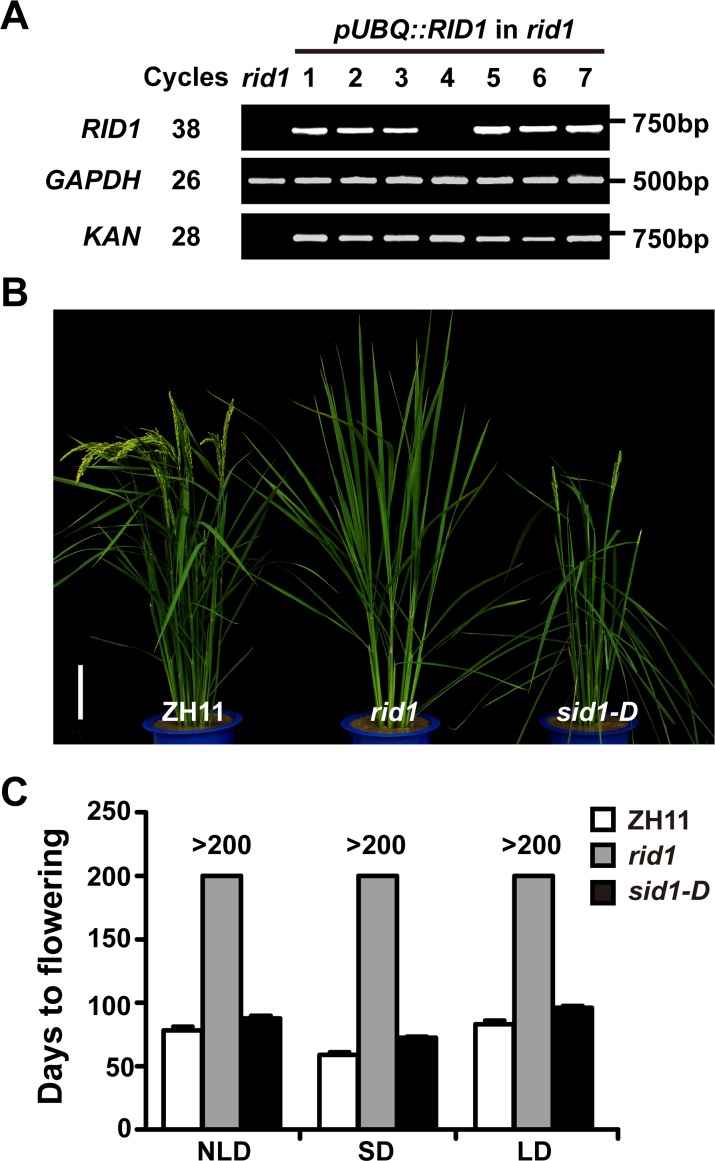
Characterization of *sid1-D*, a dominant genetic suppressor of *rid1*. (A) Transcript analyses of *RID1* in transgenic flowering lines carrying *pUBQ*::*RID1* in *rid1* background (lanes 1 to 7). Note that no transcript of *RID1* was detected in line 4 (renamed as *sid1-D*). *rid1* served as the negative control, and *GAPDH* gene served as an internal control. *KAN* amplified from *Kanamycin* gene indicated the T-DNA insertion. *pUBQ*, maize *Ubiquitin* promoter. (B) Phenotypic comparison of ZH11 (control), *rid1*, and *sid1-D* plants at the heading stage. Scale bar, 15 cm. (C) Flowering times of ZH11, *rid1*, and *sid1-D* under the indicated day length conditions (*n* = 10). NLD, natural-long-day; SD, short-day; LD, long-day conditions.

Our previous study identified a *RID1* knockout mutant (*rid1*), which shows a never-flowering phenotype under LD or SD conditions [26]. Further examination showed that T-DNA insertion at the second intron of the *RID1* gene caused the never-flowering phenotype, and a 5.7-kb genomic fragment containing the entire *RID1* coding region and its promoter could successfully rescue the mutant phenotype [26]. To examine whether the full-length cDNA of *RID1* could rescue the mutant phenotype, we generated genetic complementary plants transformed with construct (*pUBQ*::*RID1*) harboring the *RID1* cDNA fragment driven by the *Ubiquitin* promoter. Among 80 independent transgenic plants, we analyzed 7 positive transgenic plants with the restored normal flowering phenotype. Surprisingly, we discovered one line (#4) in which the transcript of *RID1* was undetectable but exhibited a restored flowering (Fig 1A). Its progeny of 200 plants exhibited a phenotypic segregation of flowering to never-flowering of 3:1 (143:57, χ^2^ = 1.13, P < 0.05), in which all flowering plants contained the selection marker *Kanamycin* gene. This observation suggests that the restored flowering plant results from a dominant mutation of a single gene that is likely to co-segregate with a T-DNA insertion event. Thus, we designated this mutant as *suppressor of rid1-D* (*sid1-D*) (Fig 1B).

Next, we investigated the heading date of *sid1-D* compared to wild-type plants. Under natural-long-day (NLD) conditions during the growing season at Wuhan, China, the heading date of *sid1-D* was delayed 10 days compared with the wild type. In the growth control room, the heading date of *sid1-D* (72.4 ± 1.1 days for SD; 101.1 ± 1.4 days for LD) was delayed about 2 weeks compared with the wild type under SD or LD conditions (Fig 1C). Furthermore, *sid1-D* exhibited a similar leaf emergence rate as that of *rid1* under both SD and LD conditions (S1A Fig). The heterozygotes and homozygous *sid1-D* exhibited an indistinguishable heading date under distinct day length conditions (S1B Fig). These results indicate that *sid1-D* is a dominant mutant that partially rescued the never-flowering phenotype of *rid1*.

### *SID1* is the rice *INDETERMINATE DOMAIN 4* (*OsIDD4*) transcription factor

**Fig 2:**
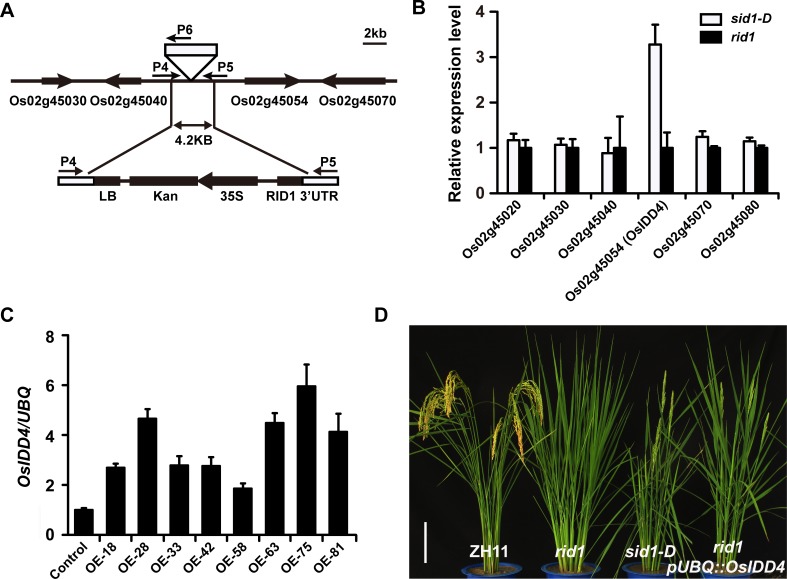
Molecular identification of *SID1*. (A) Schematic of the genomic region flanking T-DNA insertion site and T-DNA insertion region in *sid1-D*. Genes are shown as thick arrows, and intergenic regions are shown as lines at the top. T-DNA left border (LB), the selection marker *Kanamycin* driven by the *CaMV 35S* promoter, and a sequence of 3' untranslated region of *RID1* are indicated. P4 to P6 primers indicate the PCR primers used for genotyping the T-DNA in *sid1-D*. (B) Transcript analyses of the genes flanking the T-DNA in *sid1-D* and *rid1* plants. Note that the transcript of *OsIDD4* (LOC_Os02g45054) is highly elevated in *sid1-D*. Expression data relative to control were normalized to that of *Ubiquitin* (*UBQ*). Each bar represents the mean ± SEM. (C) Expression level of independent T0 transgenic flowering lines generated by transforming homozygous callus of *rid1* with *pUBQ*::*OsIDD4*. Control, transgene-negative control plant. (D) Phenotypes of wild-type (ZH11), *rid1*, *sid1-D*, and *rid1 pUBQ*::*OsIDD4* at heading stage. Scale bar, 15 cm.

Because *sid1-D* is generated by a single gene mutation and co-segregates with a T-DNA insertion, the genomic sequence flanking the left border of the T-DNA insertion site was isolated by thermal asymmetric interlaced PCR [42]. BLAST analysis of the flanking sequence indicated that a T-DNA was inserted into the intergenic region between the annotated genes LOC_Os02g45054 (*OsIDD4*) and LOC_Os02g45040 (Fig 2A). PCR analysis using the primers P1, P2, and P3 [26] indicated that the genomic background is homozygous for *rid1* (S2A Fig). We determined the genotypes of the re-introduced T-DNA insertion site by PCR amplification using the primers P4, P5, and P6 (Fig 2A and S2A Fig). All the plants homozygous or heterozygous for the T-DNA insertion showed the restored heading phenotype, whereas the plants without T-DNA insertion showed the never-flowering phenotype, like that of *rid1*. Our further analysis of the T-DNA sequence integrated into the genome indicated that a truncated T-DNA insertion event occurred. The truncated T-DNA only remained in the left region containing the selection marker *Kanamycin* driven by the *CaMV 35S* promoter and a sequence of the 3' untranslated region of *RID1* (162 bp) (Fig 2A).

Because *sid1-D* is a dominant mutant with re-introduced T-DNA inserted in the intergenic region, we examined the expression levels of genes flanking the T-DNA insertion. Quantitative reverse transcription PCR (QRT-PCR) analysis indicated that the transcript of LOC_Os02g45054 (*OsIDD4*) was significantly increased, while the other genes showed identical expression patterns in *sid1-D* and *rid1* (Fig 2B). To determine whether the elevated transcript level of *OsIDD4* is responsible for the rescued flowering in *sid1-D*, we introduced a *pUBQ*::*OsIDD4* construct (S2B Fig) into *rid1* callus. All the transgenic plants overexpressing *OsIDD4* (Fig 2C) recovered the flowering of *sid1-D* (Fig 2D), whereas plants transformed with empty vector (negative control) retained a never-flowering phenotype similar to that of *rid1* (S2C Fig). In the progenies of the rescued flowering plants, segregation of the *OsIDD4* transgene coincided very well with successful flowering, whereas the negative transgenic plants did not head, similar to *rid1* (S2C and S2D Fig). These results suggest that increased expression of *OsIDD4* is responsible for reversing the never-flowering phenotype of *rid1*. Thus, *OsIDD4* is the *Suppressor of rid1* (*SID1*).

In rice, *SID1* encodes a typical Cys-2/His-2 (C2H2) zinc finger protein belonging to the plant-specific IDD protein family, comprising 15 members in rice [28]. Phylogenetic analysis showed that SID1 belongs to a different clade than that of RID1 (S3A Fig). However, SID1 shares 43% identity with RID1; in particular, they have a highly conserved IDD at the N-terminal region (S3B Fig).

### Overexpression of *OsIDD1* or *OsIDD6* also restored flowering of *rid1*

The conserved IDD in SID1 contains four putative zinc finger domains (S3B Fig). To investigate whether the zinc fingers of SID1 are essential to complement *rid1*, we generated four constructs via ectopic expression of SID1 with mutation in each zinc finger in *rid1* (S4A Fig). A normal *SID1* CDs overexpression construct was used as a positive control. For each transformation, at least 100 independent transgenic plants were generated. The transgenic results showed that mutating each zinc finger of SID1 abolished rescuing the never-flowering phenotype of *rid1*, whereas plants transformed with normal *SID1* CDs overexpression construct recapitulated the phenotype of *sid1-D* (S4B Fig). Our results showed that the four zinc fingers of SID1 are required to restore the flowering transition in *rid1*.

**Fig 3:**
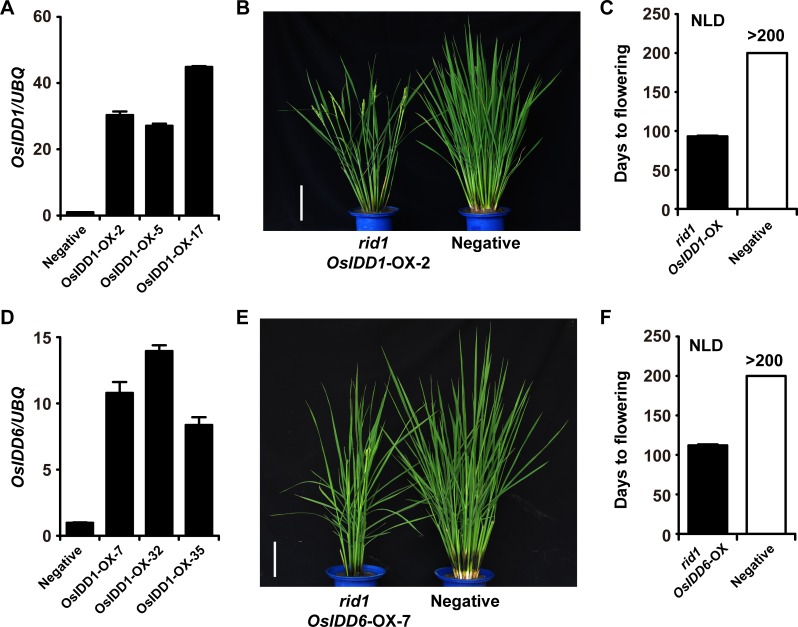
Characterization of transgenic plants overexpressing *OsIDD1* and *OsIDD6* in *rid1*. (A) Expression level of independent T0 transgenic flowering lines generated by transforming homozygous callus of *rid1* with *pUBQ*::*OsIDD1*. Negative, transgene-negative control plant. (B) Phenotypes of transgene-negative control plant and *rid1 pUBQ*::*OsIDD1* at heading stage. Scale bar, 15 cm. (C) Days to flowering under natural-long-day (NLD) conditions. Black boxes, *rid1 pUBQ*::*OsIDD1* transgenic plants; empty boxes, transgene-negative control plant (*n* = 10). (D) Expression level of independent T0 transgenic flowering lines generated by transforming homozygous callus of *rid1* with *pUBQ*::*OsIDD6*. Negative, transgene-negative control plant. (E) Phenotypes of transgene-negative control plant and *rid1 pUBQ*::*OsIDD6* at heading stage. Scale bar, 15 cm. (F) Days to flowering under NLD conditions. Black boxes, *rid1 pUBQ*::*OsIDD6* transgenic plants; empty boxes, transgene-negative control plant (*n* = 10).

Phylogenetic analysis showed there are 15 identifiable IDD genes in rice (S3A Fig). To investigate the possible redundancy of the *OsIDD* genes with *SID1*, we generated transgenic plants overexpressing the OsIDD genes *OsIDD1*, *OsIDD3*, *OsIDD6*, *OsIDD10*, *OsIDD12*, and *OsIDD14*, respectively. At least 100 independent transgenic plants of each transformation were generated. The overexpression of *OsIDD1* or *OsIDD6* in *rid1* recapitulated the phenotype of *sid1-D* plants, showing restored flowering of *rid1* (Fig 3). However, plants transformed with other *OsIDD* genes retained a never-flowering phenotype similar to *rid1*. These results suggested that *OsIDD1* and *OsIDD6* might have redundant function in floral transition with *SID1* when they were overexpressed.

### Expression patterns of *SID1* and its transcriptional activity

**Fig 4:**
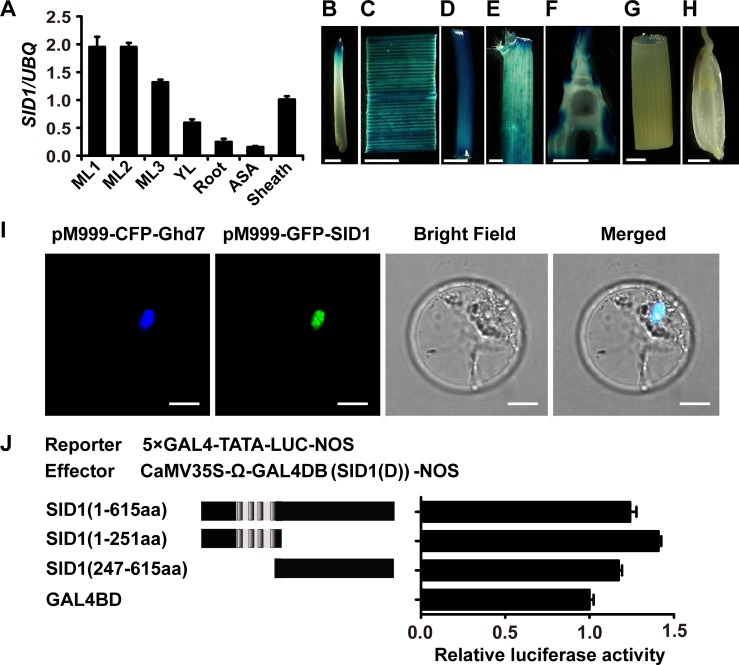
Expression patterns of *SID1* and its transcriptional activation. (A) Transcript levels of *SID1* in the indicated organs at seedling stage. The data are means ± SEM of three independent experiments. (B–H) GUS staining (blue) of distinct organs in *pSID1*::*GUS* transgenic plants: (B) root; (C) expanding leaf; (D) young leaf; (E) sheath; (F) longitudinal section of the shoot apical meristem; (G) stem; (H) floret. Scale bar, 2 mm. (I) Subcellular localization of SID1. Full-length SID1 fused with green fluorescent protein (GFP); the nuclear protein Ghd7 fused with cyan fluorescent protein (CFP) served as a nuclear marker. The two proteins were co-expressed in rice protoplasts. A bright-field image and the merged image are shown on the right. Scale bar, 10 μm. (J) Relative luciferase activities of rice protoplasts co-transfected with reporter and distinct effectors. Schemes of deletion mutants of SID1 are shown at left. Gray bars in the N-terminal region of SID1 indicate four zinc finger motifs. All luciferase activities are expressed relative to the value of GAL4 BD alone. Values represent means of three independent experiments.

To determine the spatial expression profile of *SID1*, we examined the expression level of *SID1* in various tissues by qRT-PCR at seedling stage (S5A Fig). The analysis showed that *SID1* was preferentially expressed in vegetative tissues (Fig 4A). We also made a construct *pSID1*::*GUS* and generated transgenic plants to precisely examine *SID1* expression patterns. GUS staining was detected in mature leaves, young leaves, sheath, and root tips and was most abundant in mature leaves and young leaves (Fig 4B to 4H). To examine *SID1* expression during the vegetative stage, we harvested young and expanding leaves from wild-type plants every 5 days from day 15 until floral transition. *SID1* showed an expression pattern similar to that of *RID1*, with continual expression in all examined points of young leaves (S5B Fig). In expanding leaves, the transcription level of *SID1* was higher than that of *RID1* in less than 30-day-old seedling, and then they decreased gradually during the remaining vegetative stage (S5C Fig). Like *RID1*, the expression of *SID1* did not show a diurnal expression pattern under either SD or LD conditions (S5D Fig). This expression of *SID1* and *RID1* in leaf blades indicated that their roles in flowering control for reproductive transition in rice.

Considering that *SID1* encodes a C2H2-type zinc finger transcription factor, we also assayed the subcellular localization of SID1. The construct *35S*::*SID1*::*GFP* was transiently transformed into rice protoplasts. The *SID1-GFP* exclusively co-localized with the *Ghd7-CFP* fusion protein (an established nuclear marker; [43]) (Fig 4I), indicating that SID1 is localized in the nucleus. We further examined the transcriptional activity of *SID1* in rice protoplasts using a dual luciferase reporter (DLR) assay system. All fragments of SID1, especially its N terminus (amino acids 1-251aa) enhanced the relative luciferase activity compared with the GAL4 binding domain negative control (Fig 4J). These results suggest that SID1 is a nuclear protein showing transcription activation activity.

### *SID1* is required for normal heading date in rice

**Fig 5:**
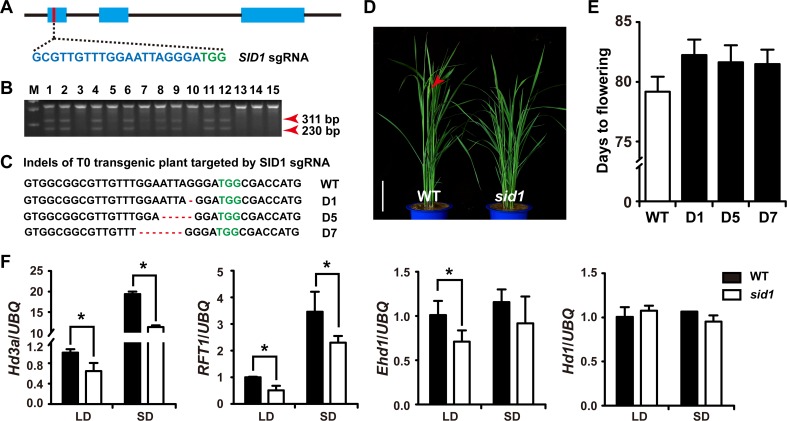
Generation and analysis of *sid1* mutants. (A) Sites within a non-conserved region of the first exon of *SID1* targeted by the CRISPR-Cas9 system. The PAM sequence (TGG) of the sgRNA target is green and the sgRNA target is cyan. (B) Outcome of *CEL*I assay to detect CRISPR-induced mutations in 15 representative T0 transgenic rice plants. Red arrowheads indicate the fragments digested by *CEL*I. (C) Representative sequences of mutant alleles identified from transgenic plants of the *SID1* sgRNA target. D1, deletion of 1 bp; D5, deletion of 5 bp; D7, deletion of 7 bp. (D) One representative T1 transgenic plant of the *SID1* sgRNA target showing a late-flowering phenotype at heading stage. Red arrowheads indicate the panicle. Scale bar, 15 cm. (E) Flowering time of *sid1* and WT plants under NLD conditions. Three independent mutant lines (D1, D5, and D7) were used for analysis. Data are means ± SD (*n* = 40). Student’s *t*-test was applied to determine significant differences (*P* < 0.05). (F) Quantitative RT-PCR analysis of *Hd3a*, *RFT1*, *Ehd1*, and *Hd1* in *sid1* and corresponding wild type (WT) under short-day (SD) and long-day (LD) conditions. The transcript levels of each gene were normalized to the rice *UBQ* gene. Values are shown as means ± SEMs of three biological replicates and each with two technical repeats. Asterisks denote significant differences (P < 0.05, Student’s *t* test).

To examine the function of *SID1* in rice, we generated *sid1* mutants using the CRISPR-Cas9 system [44]. The construct containing the Cas9 and sgRNA targeting the first exon of SID1 was designed, and 97 transgenic plants were generated (Fig 5A). PCR amplification products containing the target region were digested by *CEL*I enzyme to detect potential mutations (Fig 5B). Confirmation of the mutations by sequencing showed that the target region had small deletions of 1–7 bp and three mutant lines were used for further analysis (Fig 5C). T1 family of 40 homozygotes for each *sid1* plants (D1, D5, D7) presented a small but statistically significant (P < 0.05) delayed flowering in NLD (82.1±1.4 days for D1; 81.6±1.4 days for D5; 81.5±1.2 days for D7) compared to the wild type (79.2±1.3 days) (Fig 5D and 5E). These results suggest that mutation of *SID1* results in late heading.

A previous study reported that the expression of *Ehd1*, *Hd3a*, and *RFT1* were suppressed in *rid1* under both SD and LD conditions [26]. Therefore, we performed qRT-PCR analysis to detect the expression levels of *Ehd1*, *Hd3a*, *RFT1*, and *Hd1* in *sid1* under SD and LD conditions (Fig 5F). *Hd1* showed an almost identical expression level in *sid1* and the wild type under both conditions, suggesting that *SID1* had no effect on the expression of *Hd1*. In *sid1*, the transcript levels of *Ehd1* were partially reduced under LD conditions. Transcript levels of *Hd3a* and *RFT1* were largely reduced in the *sid1* mutants under both conditions. These results suggest that *SID1* might be involved in flowering regulation through modulation of the expression of *Ehd1*, *Hd3a*, and *RFT1*.

To investigate the possible regulation of *Hd3a* and *RFT1* by *SID1* and *RID1*, we generated transgenic plants overexpressing *SID1* or *RID1*, respectively. The independent transgenic plants overexpressing *SID1* showed a similar heading date as that in wild type (S6A to S6C Fig). Similarly, no significant changes in heading date were detected between plants with enhanced *RID1* expression and wild-type plants (S6D to S6F Fig), although the transcript levels of *Hd3a* and *RFT1* were slightly increased in some of overexpressing plants with *SID1* or *RID1*, respectively (S6G to S6J Fig). These findings indicate that overexpression of *SID1* or *RID1* would not result in early flowering in rice.

### Gain-of-function *SID1* performs the role of flowering transition in *rid1*

**Fig 6:**
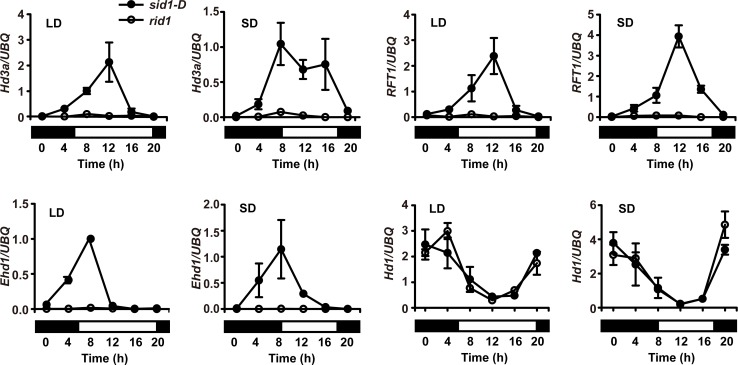
Expression patterns of flowering-related genes in *sid1-D*. The expression of *Hd3a*, *RFT1*, and *Ehd1*, but not *Hd1*, were elevated and diurnal in *sid1-D* plants under both long-day (LD) and short-day (SD) conditions. The open and filled boxes at the bottom represent the light and dark periods, respectively. The rice *UBQ* gene was used as the internal control. Values are shown as means ± SEMs of three biological replicates and each with two technical repeats.

Because overexpression of *SID1* rescued the failed flowering transition in *rid1*, we wondered whether overexpression of *SID1* would recover flowering pathways activated by *RID1*. As shown in Fig 6, the transcript levels of *Hd3a*, *RFT1*, and *Ehd1* were completely suppressed in *rid1* under either LD or SD conditions, which were the same results as in our previous investigation [26]. However, in *sid1-D* background, with the overexpression of *SID1*, the expression of *Hd3a*, *RFT1*, and *Ehd1* were elevated and diurnal under both SDs and LDs at all-time points examined during the 24 h period (Fig 6). Because *RID1* had only a slight effect on the expression of *Hd1* [26], *Hd1* showed identical expression patterns in *rid1* and *sid1-D* under both conditions (Fig 6). Thus, overexpression of *SID1* might take over the role of initiating the flowering transition in *RID1*-dependent photoperiodic flowering pathways in *sid1-D*.

**Fig 7:**
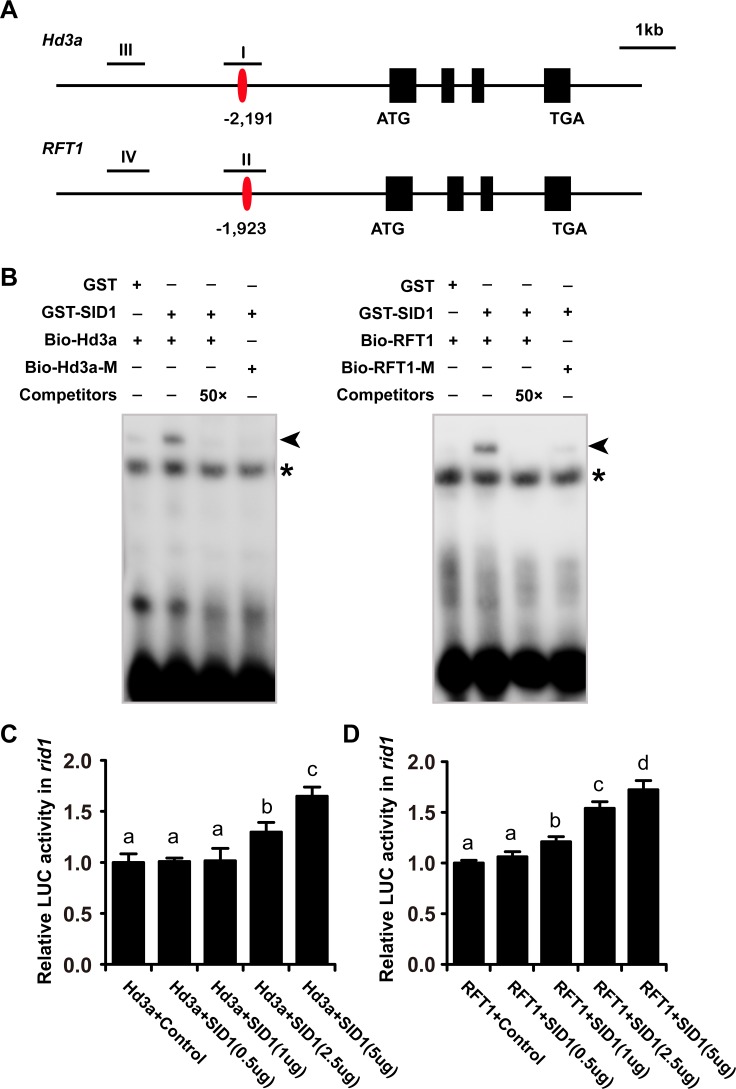
*SID1* is able to activate *Hd3a* and *RFT1* expression. (A) Schematic representation of putative loci of *SID1* binding sites in the promoter of *Hd3a* and *RFT1*. The red bars represent the core sequence containing TTTGTC. The numbers indicate the position relative to the start codon. The precipitated chromatin fragments were analyzed by qPCR using four primer sets amplifying *Hd3a* and *RFT1* regions (I, II, III, and IV) as indicated, respectively. (B) EMSA showed that SID1 could bind to the core sequence containing TTTGTC in the *Hd3a* and *RFT1* promoter *in vitro*. The *Hd3a* and *RFT1* promoter fragments containing the core sequence were incubated with GST and GST-SID1 protein *in vitro*. Unlabeled *Hd3a* and *RFT1* promoter fragments were used to compete for SID1 binding. The fragment with mutated core cis-element served as the negative control. Triangles and asterisks indicate shifted bands and nonspecific binding, respectively. (C) and (D) Ratio of firefly luciferase (LUC) to Renilla luciferase (REN) activity in *rid1* protoplasts transformed with varies dosage of SID1. Data represent means ± SDs (*n* = 4). Statistical analyses are based on a two-way analysis of variance.

Because SID1 has a highly conserved IDD belonging to members of the plant-specific zinc finger protein family, we speculated that they might exhibit the same DNA binding characteristic. Previous experiments demonstrated maize ID1 selectively binds to the consensus motifs TTTGTCG/CT/CT/aT/aT and TTTTGTCG/C by IDD *in vitro*, but the consensus motifs without T in the 5' position did not affect the binding affinity of ID1 [30]. To identify possible targets of the SID1, we surveyed the consensus motifs in the promoter regions of genes controlling flowering time in rice. As shown in Fig 7A, a core sequence containing TTTGTC was found at –2191 (I region) and –1923 (II region) in the promoter regions of *Hd3a* and *RFT1*, respectively. To examine whether SID1 could directly bind to these fragments, we performed electrophoresis mobility shift assays (EMSA) to assess the potential binding ability *in vitro*. The recombinant SID1 protein was able to bind to the fragments containing the consensus motif TTTGTC in the promoter regions of *Hd3a* or *RFT1*, respectively (Fig 7B). However, the negative controls with mutation in TTTGTC (bio-Hd3a-M or bio-RFT1-M) abolished these binding evens (Fig 7B), indicating that SID1 could specially bind to the TTTGTC motif. The results suggest that SID1 might have the ability to drive the expression of *Hd3a* and *RFT1*.

We further examined the transcriptional activity of *SID1* using a DLR assay system in *rid1* protoplasts. *Hd3a* or *RFT1* promoter driving the firefly luciferase gene was used as reporter and transfected into protoplasts of *rid1*, respectively. The construct harboring the *SID1* gene driven by *CaMV* 35S promoter was used as the effector. With the increasing effector construct containing the *SID1* gene, the luciferase activity was gradually enhanced (Fig 7C and 7D). This result further confirms that a considerable amount of SID1 has the transcriptional activation ability to drive the expression of *Hd3a* and *RFT1* when *RID1* was abolished.

### RID1 directly activates the expression of *Hd3a* and *RFT1*

**Fig 8:**
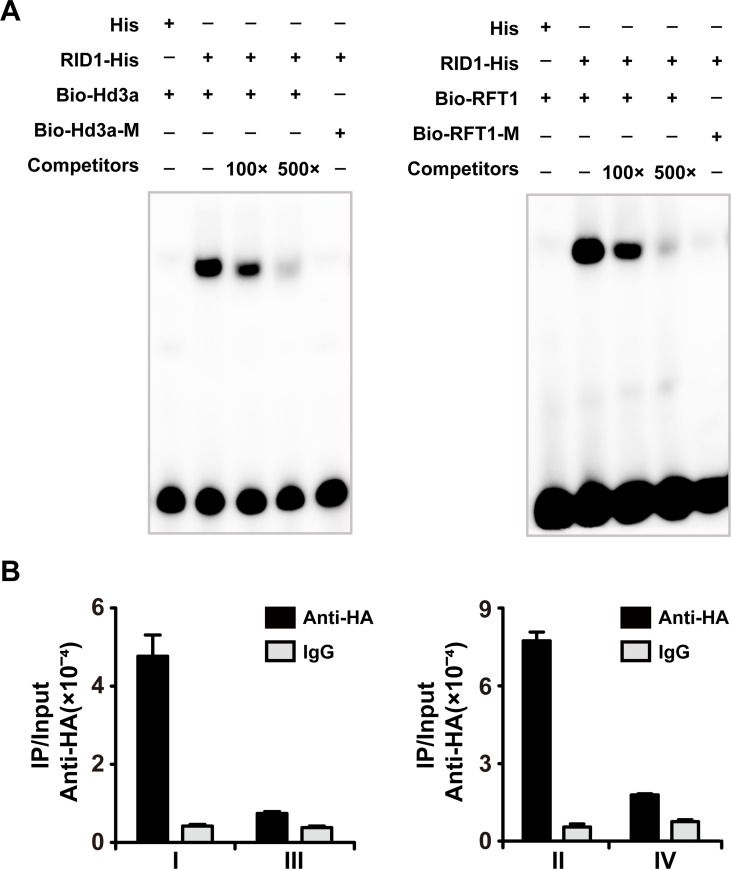
*RID1* directly bind to the promoter regions of *Hd3a* and *RFT1*. (A) Gel shift assays of His and His-RID1 recombinant proteins interacting with promoter region of *Hd3a* and *RFT1*. *Escherichia coli*–produced recombinant RID1 protein were incubated with biotin-labeled *Hd3a* and *RFT1* in the absence or presence of 100- or 500-fold molar excess of the unlabeled probes as competitor for the electrophoretic mobility shift assay (EMSA) reaction and analyzed by electrophoresis. The fragment with mutated core cis-element served as the negative control. (B) ChIP analysis of transgenic plants expressing RID1-FLAG-HA fusion protein. Nuclei from RID1-FLAG-HA transgenic plants’ leaves were immune precipitated by anti-HA. The precipitated chromatin fragments were analyzed by qPCR using four primer sets amplifying *Hd3a* and *RFT1* regions (I, II, III, and IV), as indicated in Fig 7A. The input (without antibody precipitation) chromatin was analyzed and used as the control. The ChIP experiments were repeated two times using independent biological replicates with similar results, and one representative data set is shown.

RID1 is also a member of the IDD family in rice, and QRT-PCR results demonstrated that transcription of *Hd3a* and *RFT1* were seriously reduced in the *rid1* mutant [26]. Likewise, we performed EMSA to test the potential interactions between RID1 and the promoters of *Hd3a* and *RFT1*. EMSA competition experiments demonstrated that the recombinant RID1 protein could bind to the fragments containing the consensus motif TTTGTC (Fig 8A). The binding activities of RID1 were abolished when the consensus motif was mutated to TTAATC (bio-Hd3a-M or bio-RFT1-M), indicating RID1 could specially bind to the fragments containing the consensus motif TTTGTC in the promoter regions of *Hd3a* or *RFT1*, respectively (Fig 8A). Next, we generated the construct *ProRID1*::*RID1*:*FLAG*:*HA* and introduced it into the *rid1* mutant background by *Agrobacterium*-mediated transformation. The transgenic plants successfully rescued the flowering of *rid1* (S7 Fig). Using the *ProRID1*::*RID1*:*FLAG*:*HA* transgenic plants, a chromatin immunoprecipitation (ChIP)-QPCR assays was carried out in the young leaves using HA antibody. As expected, the selected regions I and II of the *Hd3a* and *RFT1* promoters were significantly enriched in young leaves (Fig 8B). These results indicate that RID1 may initiate the flowering transition through its direct targets *Hd3a* and *RFT1*.

### Overexpressing *Hd3a* restored the flowering of *rid1*

**Fig 9:**
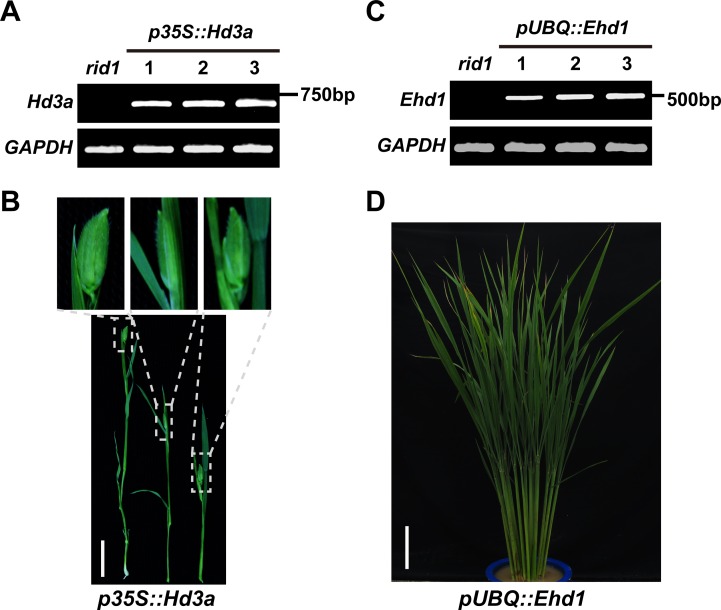
Overexpressing of *Hd3a* and *Ehd1* in *rid1* plants. (A) Expression analyses of *Hd3a* in *p35S*::*Hd3a* transgenic plants. Three independent transformed lines were analyzed. *rid1* served as the negative control. (B) *Hd3a*, driven by the *CaMV 35S* promoter (*p35S*::*Hd3a*), rescued the never-flowering phenotype of *rid1* and heading at seedling stage (T0 plants, *n* = 40). Top insets show magnifications of the panicles surrounded by dashed lines. Scale bar, 2 cm. (C) Expression analyses of *Ehd1* in *pUBQ*::*Ehd1* transgenic plants. Three independent transformed lines were analyzed. *rid1* was used as a negative control. (D) Overexpression of *Ehd1* in *rid1* (*pUBQ*::*Ehd1*) could not reverse the never-flowering phenotype of *rid1* (T0 plants, *n* = 80). All plants were grown under natural-long-day conditions. Scale bar, 15 cm.

Because the two florigen genes *Hd3a* and *RFT1* were shown to be the direct targets of RID1, we generated transgenic plants overexpressing *Hd3a* to investigate whether they can rescue the flowering transition in *rid1*. We introduced the *p35S*::*Hd3a* construct into *rid1* and obtained more than 40 transgenic plants overexpressing *Hd3a* (Fig 9A). Interestingly, all the positive transgenic plants reached flowering at the seedling stage (Fig 9B). Thus, overexpression of *Hd3a* caused early flowering in *rid1*.

Our previous investigation demonstrated that the expression of *Ehd1* and *Hd3a* were completely repressed in the *rid1* mutants [26]. Subsequently, we generated transgenic plants with overexpression of *Ehd1* in *rid1* (Fig 9C). All of the transgenic plants exhibited the never-flowering phenotype, similar to *rid1* (Fig 9D). This observation shows that overexpression of *Ehd1* is not sufficient to restore flowering transition in *rid1*. Our results further confirm that *RID1-Ehd1-Hd3a/RFT1* is not the sole pathway for floral induction mediated by *RID1* in rice [26].

## Discussion

Our previous investigation established the rough photoperiodic flowering pathway mediated by *RID1* in rice [26], but the detailed molecular mechanism of *RID1* initiating the flowering transition remained unclear. In *Arabidopsis* and yeast, dosage suppression genetic interaction has been known to apply extensively to map functional connections among genes [45–47]. To our knowledge, this is the first report to identify a suppressor functionally related to a certain gene in rice. *SID1*, a suppressor of *rid1*, was identified as a rice flowering promoter in this study. Gain of function of *SID1* led to rescue of the never-flowering phenotype in *rid1*. SID1 and RID1 showed the binding ability with the promoter regions of *Hd3a* and *RFT1* to drive expression of *Hd3a* and *RFT1*. Our new findings indicate that *RID1* and *SID1* might be involved in the autonomous flowering pathway regulating the transition to flowering in rice.

### *SID1* is a flowering promoter

Although the identification of *SID1* could be due to mere chance in the transgenic events, our genetic and molecular analyses clearly suggested that *SID1* is required for promoting flowering in rice. *SID1* encodes an IDD-type zinc finger transcription factor, preferentially expressed in mature leaves (Fig 4A), where floral inductive cues are perceived or initiated [22, 29]. Mutation in *SID1* caused delayed flowering time compared to that of the wild type (Fig 5D and 5E). Overexpression of *SID1* could successfully restore the flowering transition in *rid1* (Fig 2C and 2D). In addition, the expression levels of *Hd3a* and *RFT1* were greatly suppressed in *sid1* (Fig 5F). However, the expression of *Ehd1* was slightly reduced in *sid1* plants (Fig 5F), but was almost completely repressed in the *rid1* mutants [26], suggesting that the *Ehd1*-mediated flowering pathways may differ between *rid1* and *sid1* mutants. This observation coincides with evidence that *rid1* shows the strongest phenotype, never flowering, whereas *sid1* shows only slightly delayed flowering (Fig 5D and 5E).

*RID1* acts as a master switch for floral transition. SID1 and RID1 might exert their function in the flowering transition with RID1 having priority for driving the expression of *Hd3a* and *RFT1*. When the function of *RID1* is abolished, only the normal expressing level of *SID1* may not enough to trigger the expression of *Hd3a* and *RFT1*, or due to non-overlapping expression patterns between *SID1* and *RID1*. Thus, *rid1* plants remain in the vegetative growth stage. However, increasing or ectopic expressing *SID1* transcripts is responsible for reverting *rid1* to the phase of flowering. Subsequently, we demonstrated that florigen genes, *Hd3a* and *RFT1*, are up-regulated in *sid1-D* (Fig 6). Furthermore, SID1 binds *Hd3a* and *RFT1* promoter region *in vitro* (Fig 7B), and the LUC activity enhanced with increasing levels of SID1 in *rid1* protoplasts (Fig 7C and 7D). These evidences support the function of *SID1* recovering, at least in part, the *SID1*-*Hd3a*/*RFT1* pathway to elicit flowering when *RID1* is abolished. Expression of *Ehd1* was also elevated in *sid1-D* plants (Fig 6), suggests that other pathways regulated by *RID1* may also be activated by overexpression of *SID1*.

### Roles of IDD protein in promoting flowering transition

Both SID1 and RID1 are IDD zinc finger proteins. Proteins containing an IDD comprise a family of zinc finger transcription factors that are unique to plants [28]. The recognition of the DNA consensus sequence is likely to be mediated by the zinc finger modules located in the IDD [30]. The highly conserved IDD is composed of four putative zinc finger domains with spacer sequences between them [28, 30]. *In vitro* DNA binding experiments showed that the second and third zinc fingers in the IDD are required for interaction with the DNA consensus motif [30]. Moreover, a different spacer between these zinc fingers modules in the IDD does not alter DNA binding specificity [30]. In this study, genetic evidences suggest that overexpression of *SID1*, *OsIDD1* or *OsIDD6* could restore the *rid1* mutant to flowering successfully. We propose the function of *SID1*, *OsIDD1*, and *OsIDD6* are redundant and that overexpression any of them could take the place of *RID1* to initiate the flowering transition when *RID1* is absent. However, because mutation of any of zinc fingers of SID1 abolished rescuing the never-flowering phenotype of *rid1* (S4A and S4B Fig), suggesting that the first and fourth zinc fingers in the IDD also have critical roles in mediating DNA-protein interactions. Given that *SID1*, *OsIDD1*, and *OsIDD6* are some co-expression (S8A and S8B Fig) and *sid1* null mutants displayed moderate late flowering phenotypes (Fig 5D and 5E), future research is needed to develop the double and triple mutants to understand whether these *OsIDDs* coordinately modulate flowering time or not.

### *RID1* may participate in flowering transition through an autonomous pathway in rice

Our previous investigation indicated that *RID1* activates the expression of florigen genes (*Hd3a*, *RFT1*) mainly by regulating the expression of *Ehd1* and *Hd1* [26]. These findings suggest that *RID1* is involved in two independent photoperiod pathways, mediated by *Ehd1* and *Hd1*, respectively. However, plants harboring nonfunctional alleles of *Hd1* and *Ehd1* still flower under either SD or LD conditions [48], demonstrating that *RID1* may play a role in alternative flowering pathway(s) for the flowering transition. In this study, overexpression of *Ehd1* could not reverse the never-flowering phenotype of *rid1* (Fig 9C and 9D), further verifying this speculation.

A striking finding of our study is that *Hd3a* and *RFT1* are the direct targets of *RID1* (Fig 8). Transcription of *Hd3a* and *RFT1* was completely repressed in *rid1* [26], and genetic analysis showed that ectopic expression of *Hd3a* could reverse the never-flowering phenotype of *rid1* (Fig 9A and 9B), indicating that florigen genes indeed act downstream of *RID1*. Furthermore, ChIP investigations indicated that RID1 could bind the promoter region of *Hd3a* and *RFT1* in young leaves (Fig 8B), suggested that RID1 initiates the expression of *Hd3a* and *RFT1* most likely occurred in the early vegetative stages. Hd3a and RFT1, two florigens in rice, be synthesized in leaves and transported to the shoot apex, and induced flowering [49–51]. As previously reported, photoperiod variation, temperature, gibberellin, age, and nutrition have been implicated in floral induction [11, 52]; thus, the transition to flowering is complex and involves the convergence of multiple signals onto the florigen genes. *RID1* acts as the master switch of phase transition and may function in initiating the expression of florigen genes (*Hd3a* and *RFT1*) at the early stage, and then several floral induction cues converge to accumulate florigens to promote floral transition.

Indeed, *RID1* expression was detected most abundantly in young leaves at early seedling stages and is unaffected by photoperiod, indicating that *RID1* may regulate an autonomous signal for flowering transition. Recent transcription and metabolism analyses showed that maize *ID1* actually affects primary carbohydrate metabolism–related genes’ function and is closely associated with florigen production in maize mature leaves [39]. Although no clear ortholog of *RID1* exists in *Arabidopsis*, the *AtIDD8* was found to regulate sugar transport and metabolism contributing to photoperiodic flowering time [31]. Therefore, mediation of autonomous floral induction by *RID1* may involve coordinating the state of carbohydrate metabolism in rice. Characterizing the transcript and metabolite signature changes in *rid1* would further provide clues to help us further understand the mechanism(s) underlying the vegetative–reproductive phase transition in rice.

## Materials and methods

### Plant materials and growth conditions

The rice variety used in this study was *Oryza sativa* subsp. *japonica* ‘Zhonghua11’ (ZH11). Plants were grown under NLD conditions in the experimental field during the rice growing season of Huazhong Agriculture University in Wuhan, China, and in a greenhouse during the winter. All transgenic plants were grown under similar growth conditions. Plants were grown in controlled-growth chambers (Conviron) under SD (10 h light at 26°C/14 h dark at 24°C) or LD (14 h light at 26°C/10 h dark at 24°C) conditions with a relative humidity of 70%. The light intensity was 800 μmol m^-2^ s^-1^.

### Plasmid construction and rice transformation

To generate *pUBQ*::*OsIDDs* transgenic plants, the *OsIDD* genomic DNA sequence was amplified and then cloned into pU2301, which was modified from pC2301 vector with the maize *Ubiquitin* promoter, and then verified by sequencing. An empty pU2301 vector was used as a negative control. For overexpression of *Ehd1*, the *Ehd1* genomic DNA sequence was amplified with primer pair Ehd1-OX-F/Ehd1-OX-R and then cloned into pU2301 by *Kpn*I-*BamH*I digestion. For overexpression of *Hd3a*, the *Hd3a* genomic DNA sequence was amplified with primer pair Hd3a-OX-F/Hd3a-OX-R and then cloned into pS2300 by *Xba*I-*Kpn*I digestion. To obtain *ProRID1*::*RID1*:*FLAG*:*HA* transgenic plants, genomic fragments containing the *RID1* promoter and coding region lacking a stop codon were amplified with primer pair PFA2300-RID1-F/PFA2300-RID1-R and then cloned in frame into pFA2300 (kindly provided by Saifeng Cheng, Huazhong Agricultural University) by *Kpn*I digestion. The constructs were introduced into *Agrobacterium tumefaciens* EHA105 and homozygous callus from *rid1* was used as the transformation recipient.

To generate *pUBQ*::*SID1(cDNA)* and *pUBQ*::*RID1(cDNA)* transgenic plants, the coding sequences were amplified by RT-PCR and ligated into the pEASY-T3 vector (TransGen Biotech), and then verified by sequencing. The resulting plasmids were used as templates. Full-length cDNA of *SID1* were amplified with primer pair SID1(CDs)-OX-F/SID1(CDs)-OX-R and then cloned into pU2301 by *Kpn*I-*BamH*I digestion; full-length cDNA of *RID1* was amplified with primer pair RID1(cDNA)-OX-F/RID1(cDNA)-OX-R and then cloned into pU2301 by *Kpn*I-*BamH*I digestion. To investigate the tissue-specific expression of *SID1*, approximately 3-kb promoter fragments of *SID1* were amplified from genomic DNA and then cloned into pC2300-EX-GUS [53] to create *pSID1*::*GUS*. To introduce targeted mutations in SID1 protein, sgRNA:Cas9 expression vector of the *SID1* gene was constructed as described previously [44]. The constructs were introduced into *A*. *tumefaciens* EHA105 and transformed into the callus derived from ZH11. All primers used for genotyping and vector construction are listed in S1 Table.

### Site-directed mutagenesis

Site-directed mutagenesis was introduced by three-step PCR. Full-length *SID1* CDs in pEASY-T3 vector were used as templates in the first and second PCR amplifications. In the first PCR, the forward primer SID1(CDs)-OX-F and reverse primers containing the desired mutation were used. In the second PCR, the forward primers containing the desired mutation, which was the complement sequence of the first PCR reverse primer, and reverse primer SID1(CDs)-OX-R were used. The first and second PCR products were purified, and this mixture was used as a template for the final PCR amplification with primers SID1(CDs)-OX-F/SID1(CDs)-OX-R. The final products were inserted into the pU2301 vector and confirmed by sequence analyses. The resulting plasmids were introduced into *A*. *tumefaciens* EHA105 and homozygous callus from *rid1* was used as the transformation recipient. All primers for site-directed mutagenesis are listed in S1 Table.

### Identification mutants by CRISPR-Cas9 system

Genomic DNA from individual transgenic plants was extracted for PCR analysis. The *CEL*I assay was used to identify the potential mutations. The PCR products amplified with SID1-specific primers SID1-CE-F/SID1-CE-R from individual mutant plants were cloned into pEASY-T3 vector (TransGen Biotech) for sequencing.

### GUS assay

GUS staining and imaging were carried out as described previously [26].

### RT-PCR and qRT-PCR analyses

Total RNA was extracted using TRIzol reagent (Invitrogen). RNA (2 μg) was treated with RNase-free DNaseI (Invitrogen), and first-strand cDNA was synthesized by M-MLV reverse transcriptase (Invitrogen) in a volume of 150 μl. For RT-PCR analysis, 3 μl of the first-strand cDNA described above was used as a template for PCR in a reaction volume of 20 μl. *GAPDH* served as a control for mRNA levels. qRT-PCR was run in a total volume of 10 μl containing 4.4 μl of the reverse-transcribed product described above, 0.3 μM gene-specific primers, and 5 μl FastStart Universal SYBR Green Master (Rox) superMIX (Roche) on an Applied Biosystems ViiA 7 Real-Time PCR system or the ABI PRISM 7500 sequence detection system according to the manufacturer’s instructions. Rice *Ubiquitin* was set as an internal control. The measurements were obtained using the relative quantification method. All primers are listed in S1 Table.

### Subcellular localization of SID1

To construct the subcellular localization plasmids, the full-length CDs of *SID1* were amplified with primers SID1-pM999-F and SID1-pM999-R with *EcoR*I-*Kpn*I digestion sites and inserted into pM999-GFP for fusion with the reporter gene. Rice protoplasts were isolated from 13-day-old etiolated seedlings and transformed with the tested pairs of constructs. Fluorescence in the transformed protoplasts was imaged using a confocal laser scanning microscope (TCS SP2; Leica) after incubation at 23°C for 12–16 h.

### Transcriptional activity analysis

The transcriptional activity of *SID1* was analyzed using the DLR assay system in rice protoplasts prepared from etiolated seedlings [54]. The firefly luciferase gene driven by the minimal TATA box of the *CaMV 35S* promoter following five copies of the GAL4 binding element was used as a reporter. The *Renilla luciferase* gene driven by *CaMV 35S* was used as an internal control. The different deletion fragments of SID1 were amplified and then fused with the yeast GAL4 DNA-binding domain as effectors, driven by *CaMV 35S* followed by the translational enhancer Ω from tobacco mosaic virus. For each assay, 2.5 μg reporter plasmid DNA, 2.5 μg effector plasmid DNA, and 0.5 μg internal control plasmid DNA were co-transfected. After incubating for 12–16 h at 23°C, the relative luciferase activity was measured using the DLR assay system and the TECAN Infinite M200 microplate reader.

To assess the specific binding and activity of *Hd3a* and *RFT1* promoters, protoplasts were prepared from 2-week-old fully green tissue of *rid1* [55]. The coding sequence of *SID1* was amplified and then fused into the NONE vector, an effector plasmid driven by the *CaMV 35S* promoter followed with the translational enhancer Ω sequence. To generate the *Hd3a*::*LUC* and *RFT1*::*LUC* reporter genes, the *Hd3a* and *RFT1* promoters were amplified (specific primers are listed in S1 Table) and inserted into 190-LUC vector, respectively. The *Renilla luciferase* gene driven by *CaMV 35S* was used as a normalization control. The recovery assays were performed with *Hd3a*::*LUC* or *RFT1*::*LUC* plus 35S::SID1 at various dosages, respectively.

### Electrophoretic mobility shift assays

To express the RID1 protein in *Escherichia coli*, the CDs of RID1 were amplified with primers 32a-RID1-F and 32a-RID1-R cloned into the *BamH*I-*EcoR*I sites of the pET-32a expression vector (Novagen) and then introduced into Transetta (DE3) cells (TransGen Biotech). The target protein was purified with Ni-NTA agarose (Qiagen). To express the SID1 protein in *E*. *coli*, the CDs of SID1 were amplified with primers pGEX-4T-SID1-F and pGEX-4T-SID1-R cloned into the *BamH*I-*EcoR*I sites of the pGEX-4T-1 expression vector (GE Healthcare) and then introduced into Transetta (DE3) cells (TransGen Biotech). The target protein was purified with GST Fast Flow (GE Healthcare).

The *Hd3a* promoter (including the consensus motif TTTGTC), *Hd3a-M* promoter (with nucleotide TTAATC replacement in the consensus motifs), *RFT1* promoter (including the consensus motif TTTGTC), and *RFT1-M* promoter (with nucleotide TTAATC replacement in the consensus motifs) were produced by annealing of oligonucleotides with biotin 5'-end labeled *Hd3a-EMSA-F/R*, *Hd3a-EMSA-MF/MR*, *RFT1-EMSA-F/R*, and *RFT1-EMSA-MF/MR*, respectively. For each reaction, 50 fmol biotin-labeled probes were incubated with the His-RID1 or GST-SID1 protein in the binding buffer (10 mM Tris, 50 mM KCl, 10 μM ZnCl_2_, 1 mM DTT, 1 μg/μl poly(dI-dC), 0.1% BSA, 2.5% glycerol, and 0.05% NP-40) for 30 min on ice using the LightShift Chemiluminescent EMSA kit. After incubation, the DNA–protein complex was separated by 6% native polyacrylamide gel electrophoresis. After separation, the signal of biotin was developed using the Chemiluminescent Nucleic Acid Detection Module (Thermo, USA) according to the manufacturer’s protocol. Images were visualized on Tanon-5200 Chemiluminescent Imaging System (Tanon Science and Technology).

### Chromatin co-immunoprecipitation assay

Chromatin co-immunoprecipitations (ChIPs) were performed as described previously [56]. In brief, the young leaves of *ProRID1*::*RID1*:*FLAG*:*HA* transgenic plants were fixed in formaldehyde in a vacuum. The chromatin solution was sonicated and the soluble chromatin fragments were obtained from isolated nuclei. Pre-adsorption with Dynabeads protein G (Invitrogen) was performed to remove nonspecific binding DNA. Immunoprecipitation with anti-HA specific antibody (Pierce HA Tag IP/Co-IP; #26180) and IgG were performed as described previously [56]. Immunoprecipitated DNA was analyzed by qRT-PCR, and the primers are listed in S1 Table online.

## Supporting information

S1 Fig Characterization of phenotypes of *sid1-D*. (A) Comparison of leaf emergence rates between *rid1* and *sid1-D* plants under both short-day (SD) and long-day (LD) conditions during development (mean ± SD, *n* = 8). Arrow indicates the flowering time of *sid1-D* plants. (B) Flowering time of *sid1-D* and heterozygote (HETE) plants under distinct day length conditions (*n* = 10). NLD, natural long day.
(TIF)

S2 Fig Genotyping and overexpression of *OsIDD4* in *rid1* mutant. (A) Linkage analysis of the T-DNA and *sid1-D* phenotype. P1 to P3 primers, which were described by Wu et al. [26], were used to ensure the tested plants are in the *rid1* mutant background. P4 to P6 primers indicate the PCR primers used for genotyping the re-introduced T-DNA in *sid1-D*. (B) Schematic representation of the construct used for overexpression of *OsIDD4*; the construct was named *pUBQ*::*OsIDD4*. (C) Plants transformed with empty vector (negative control) retained a never-flowering phenotype similar to that of *rid1*. All positive transgenic T1 plants (left) derived from a transgenic T0 line can flower normally, whereas all negative segregants never flowered (right). Scale bar, 15 cm. (D) Co-segregation between flowering time and the transgenic fragment in T1 segregants derived from a single copy restored line (T0).
(TIF)

S3 Fig Phylogenetic analysis of the INDETERMINATE DOMAIN (IDD) family genes and alignment of deduced amino acid sequences of SID1 and RID1 in rice. (A) A phylogenetic tree of rice IDD family proteins based on IDD domain sequences. Phylogenetic analysis was conducted using MEGA 5.1. Fifteen IDD proteins were selected for establishing a bootstrap neighbor-joining phylogenetic tree and 1000 replicates were conducted to determine the statistical support for each node. (B) Alignment of amino acid sequences of RID1and SID1 proteins. The identical amino acids are shown with white text on a black background. Underlines show the position of putative zinc finger domain (ID domain). The putative nuclear localization signal motifs (NLS) are shown by blue bars.
(TIF)

S4 Fig Analysis of the DNA binding function of each putative zinc finger motif of SID1. (A) Schematic diagram of mutant SID1 protein used in transgenic experiments. Structure of the ID domain to SID1 is shown on top. Zinc fingers (Z1 to Z4) are indicated as colored boxes. C and H indicate cysteines and histidines that define putative zinc fingers. Numbers indicate the amino acid position of C and H residues of the SID1 protein. Zinc fingers were disrupted by replacing the first cysteine pair of each module with two alanine residues: Z1M (C97A, C100A), Z2M (C139A, C144A), Z3M (C174A, C177A), and Z4M (C201A, C203A) represent ID domain proteins with mutant versions of Z1, Z2, Z3, and Z4, respectively. X indicates each putative zinc finger was disrupted. (B) Mutating each zinc finger of SID1 could not rescue the never-flowering phenotype of *rid1*. A normal SID1 CDs overexpression plant served as the positive control. Scale bar, 15 cm.
(TIF)

S5 Fig Developmental and rhythmic expression of *SID1*. (A) 35-day-old wild-type plants (Zhonghua 11) grown under natural-long-day (NLD) conditions were used for qRT-PCR. ML1, newly emerging leaf; ML2, expanding leaf; ML3, fully expanded leaf; ML1, ML2, and ML3 collectively referred to as mature leaf (ML); YL, young leaf; ASA, around the shoot apex. Scale bar, 15 cm. (B) and (C) Expression analyses of *RID1* and *SID1* in young and mature leaves under NLD conditions during vegetative stage. (D) Rhythmic expression of *SID1*. The rice *Ubiquitin* (*UBQ*) gene served as the internal control. Values are shown as means ± SEMs of three independent experiments. The open and filled bars at the top represent the light and dark periods, respectively.
(TIF)

S6 Fig Characterization of *SID1*- and *RID1*-overexpressing plants. (A) Transcript analyses of *SID1* in SID1-OX lines. Samples were harvested from 35-day-old plants under natural-long-day (NLD) conditions. *Ubiquitin* served as a control. Values are means ± SEMs of three replicate samples. (B) Days to flowering under long-day (LD, left) and short-day (SD, right) conditions. Black boxes, segregating wild type (WT); empty boxes, SID1-overexpressing lines (*n* = 10). (C) Phenotypes of segregating WT (left) and SID1-OX (right) plants at heading stage. Scale bar, 15 cm. (D) Transcript analyses of *RID1* in RID1-OX lines. Samples were harvested from 35-day-old plants under NLD conditions. *Ubiquitin* served as a control. Values are means ± SEMs of three replicate samples. (E) Days to flowering under NLD conditions. Black boxes, segregating WT; empty boxes, SID1-overexpressing lines (*n* = 10). (F) Phenotypes of segregating WT (left) and RID1-OX (right) plants at heading stage. Scale bar, 15 cm. (G–J) Quantitative RT-PCR analysis of *Hd3a* and *RFT1* in *SID1* and *RID1* overexpressing plants under NLD conditions. The transcript levels of each gene were normalized to the rice *UBQ* gene. Values are shown as means ± SEMs of three independent experiments.
(TIF)

S7 Fig Characterization of *ProRID1*::*RID1*:*FLAG*:*HA* transgenic plants. (A) Phenotypes of *rid1* and *ProRID1*::*RID1*:*FLAG*:*HA* transgenic plants at heading stage. Scale bar, 15 cm. (B) Co-segregation between flowering time and the transgenic fragment in T1 segregants derived from a single copy restored line (T0). (C) Protein level of *RID1* in *rid1* and *ProRID1*::*RID1*:*FLAG*:*HA* transgenic plants.
(TIF)

S8 Fig Expression patterns of *OsIDD1* and *OsIDD6*. (A) Transcript levels of *OsIDD1* in the indicated organs (S5A Fig). The data shown are the means ± SEMs of three independent experiments. (B) Transcript levels of *OsIDD6* in the indicated organs. The data shown are the means ± SEMs of three independent experiments.
(TIF)

S1 Table Primers used in this study. (DOCX)
