# Supplementary figures and images for: Suppressor of rid1 (SID1) shares common targets with RID1 on florigen genes to initiate floral transition in rice
Source: PLoS Genet. 2017 Feb 24;13(2):e1006642. doi: 10.1371/journal.pgen.1006642 (PMC5345856; doi:10.1371/journal.pgen.1006642)

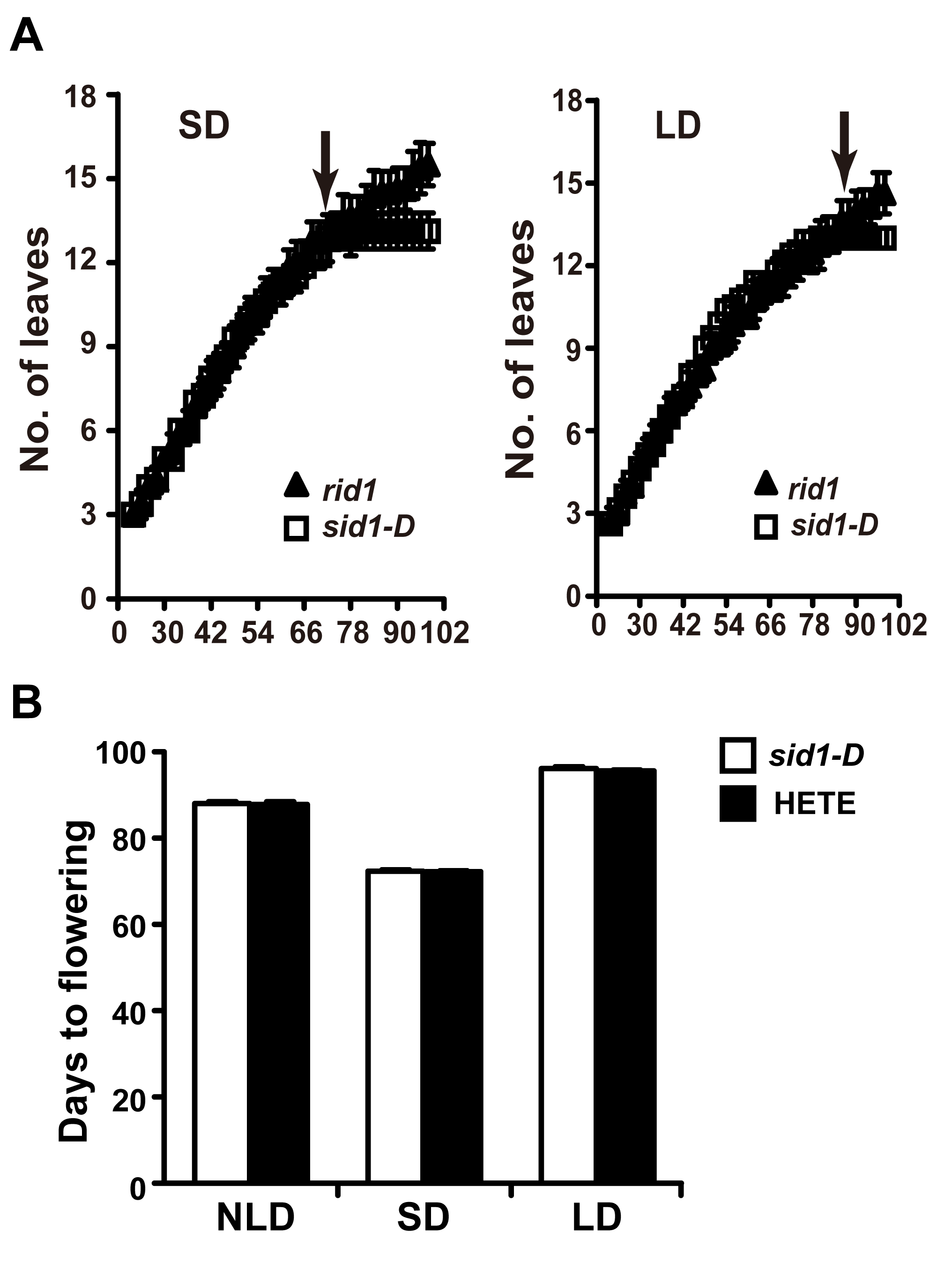

Supplement: S1 Fig — (A) Comparison of leaf emergence rates between rid1 and sid1-D plants under both short-day (SD) and long-day (LD) conditions during development (mean ± SD, n = 8). Arrow indicates the flowering time of sid1-D plants. (B) Flowering time of sid1-D and heterozygote (HETE) plants under distinct day length conditions (n = 10). NLD, natural long day. (TIF) [file pgen.1006642.s001.tif]

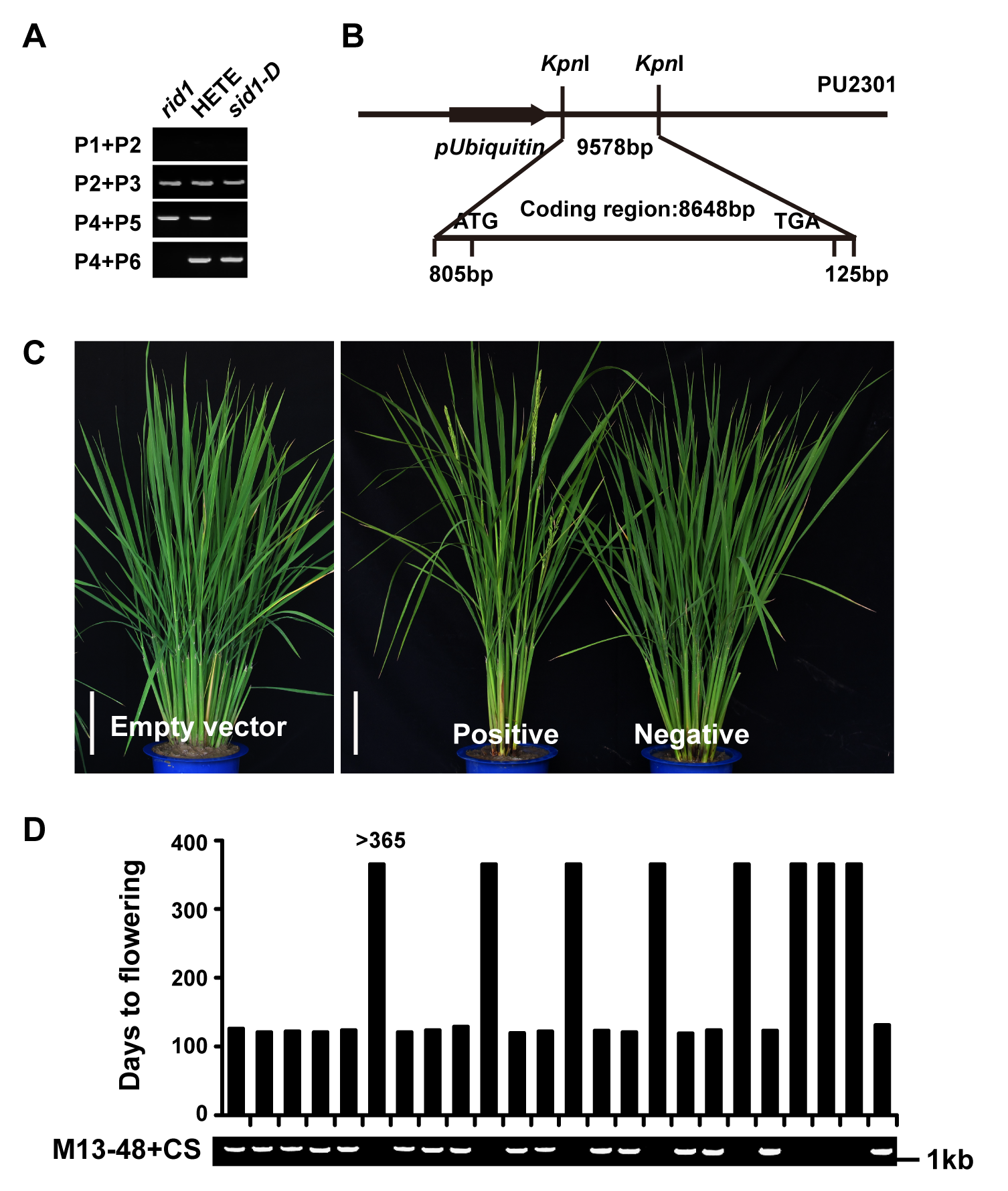

Supplement: S2 Fig — (A) Linkage analysis of the T-DNA and sid1-D phenotype. P1 to P3 primers, which were described by Wu et al. [26], were used to ensure the tested plants are in the rid1 mutant background. P4 to P6 primers indicate the PCR primers used for genotyping the re-introduced T-DNA in sid1-D. (B) Schematic representation of the construct used for overexpression of OsIDD4; the construct was named pUBQ::OsIDD4. (C) Plants transformed with empty vector (negative control) retained a never-flowering phenotype similar to that of rid1. All positive transgenic T1 plants (left) derived from a transgenic T0 line can flower normally, whereas all negative segregants never flowered (right). Scale bar, 15 cm. (D) Co-segregation between flowering time and the transgenic fragment in T1 segregants derived from a single copy restored line (T0). (TIF) [file pgen.1006642.s002.tif]

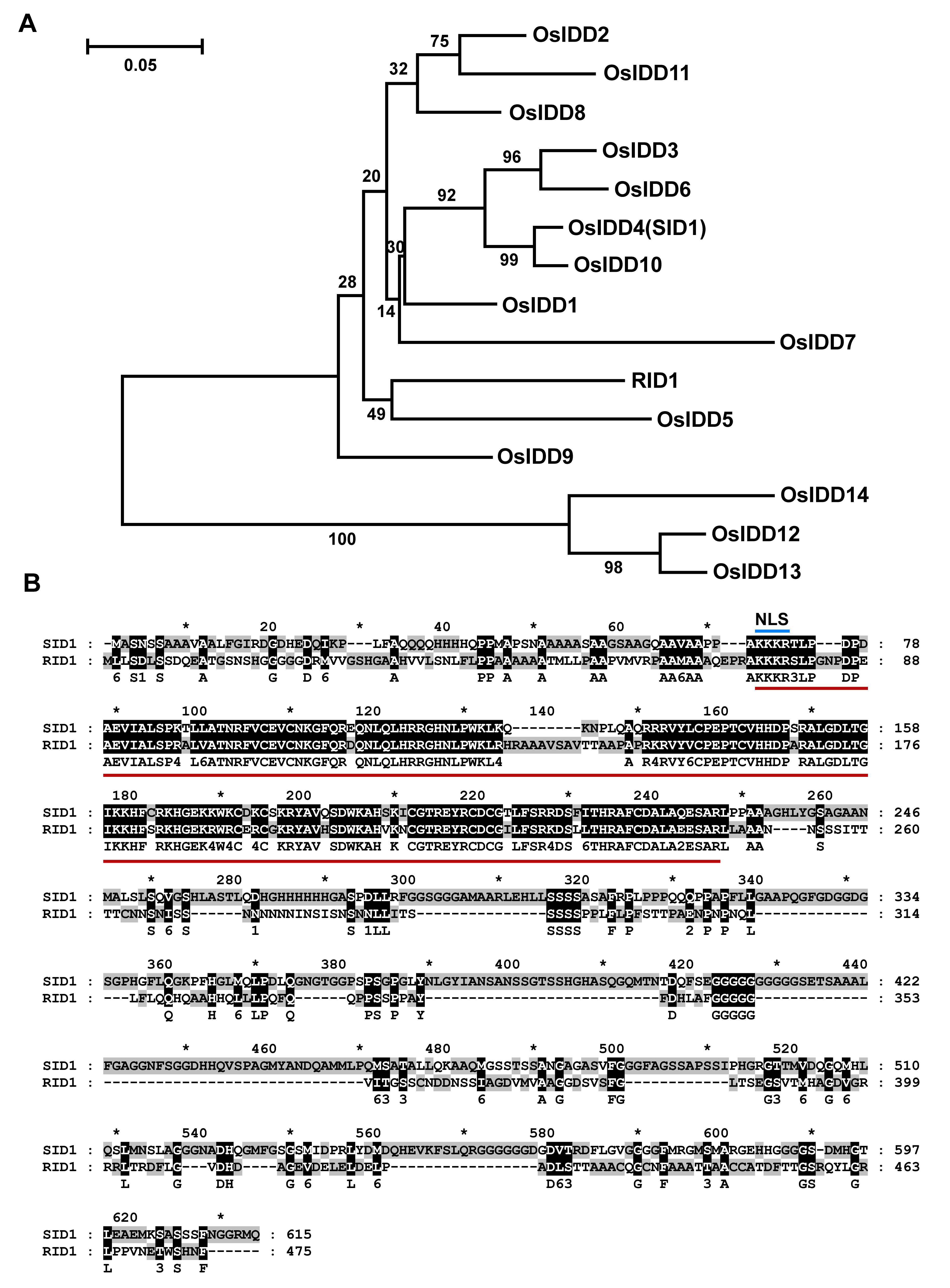

Supplement: S3 Fig — (A) A phylogenetic tree of rice IDD family proteins based on IDD domain sequences. Phylogenetic analysis was conducted using MEGA 5.1. Fifteen IDD proteins were selected for establishing a bootstrap neighbor-joining phylogenetic tree and 1000 replicates were conducted to determine the statistical support for each node. (B) Alignment of amino acid sequences of RID1and SID1 proteins. The identical amino acids are shown with white text on a black background. Underlines show the position of putative zinc finger domain (ID domain). The putative nuclear localization signal motifs (NLS) are shown by blue bars. (TIF) [file pgen.1006642.s003.tif]

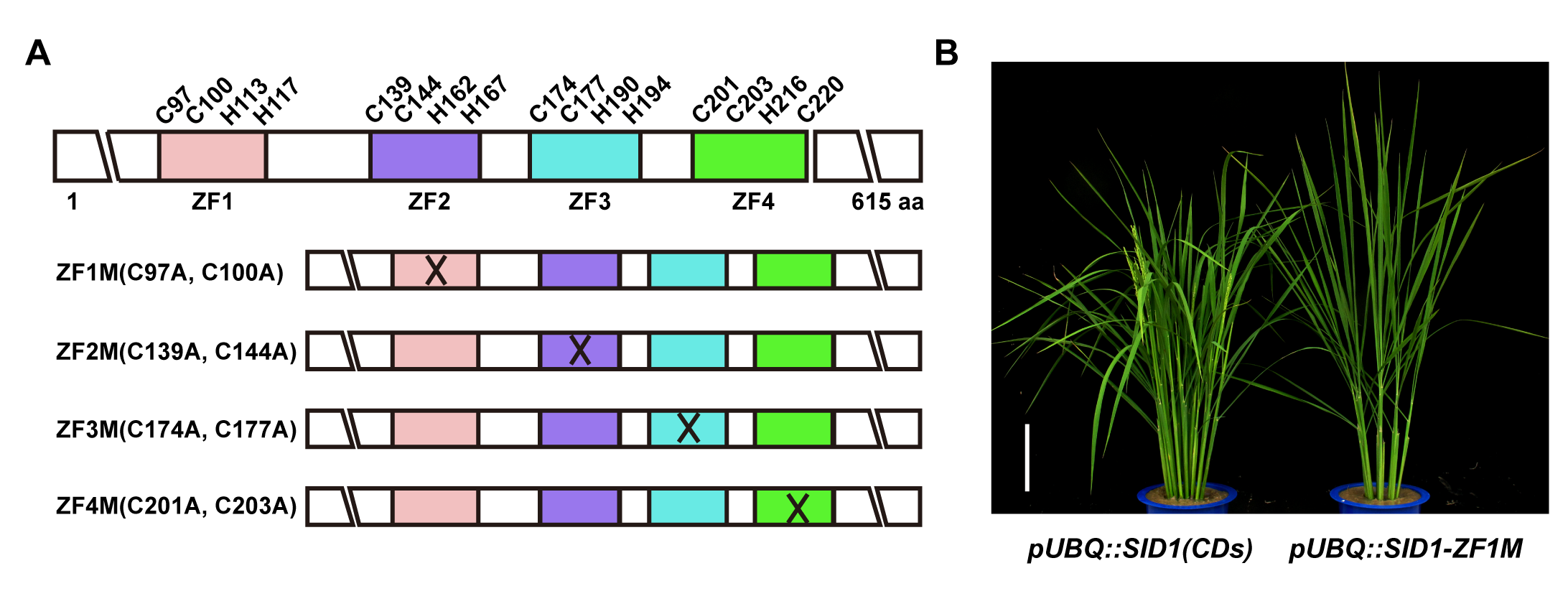

Supplement: S4 Fig — (A) Schematic diagram of mutant SID1 protein used in transgenic experiments. Structure of the ID domain to SID1 is shown on top. Zinc fingers (Z1 to Z4) are indicated as colored boxes. C and H indicate cysteines and histidines that define putative zinc fingers. Numbers indicate the amino acid position of C and H residues of the SID1 protein. Zinc fingers were disrupted by replacing the first cysteine pair of each module with two alanine residues: Z1M (C97A, C100A), Z2M (C139A, C144A), Z3M (C174A, C177A), and Z4M (C201A, C203A) represent ID domain proteins with mutant versions of Z1, Z2, Z3, and Z4, respectively. X indicates each putative zinc finger was disrupted. (B) Mutating each zinc finger of SID1 could not rescue the never-flowering phenotype of rid1. A normal SID1 CDs overexpression plant served as the positive control. Scale bar, 15 cm. (TIF) [file pgen.1006642.s004.tif]

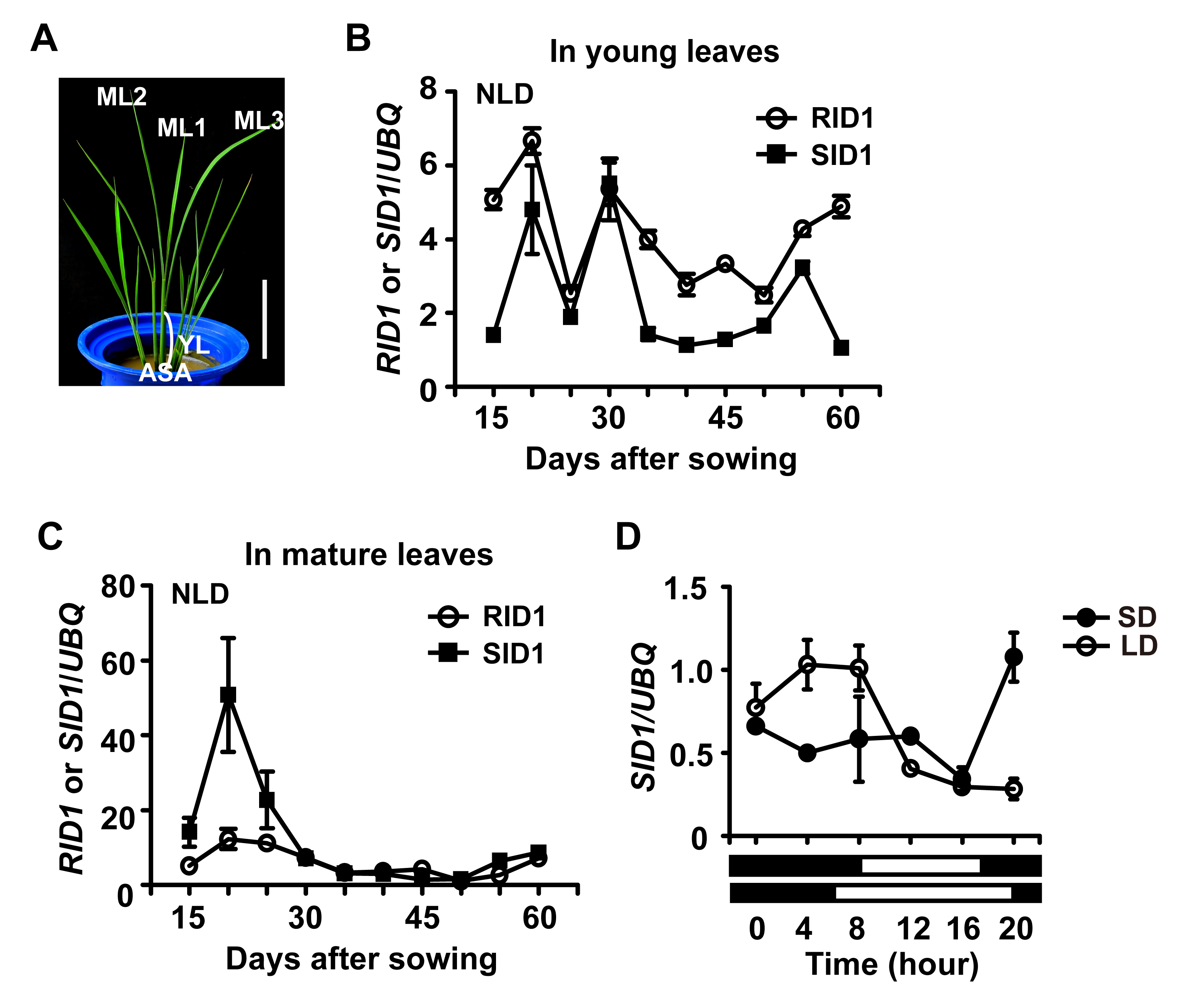

Supplement: S5 Fig — (A) 35-day-old wild-type plants (Zhonghua 11) grown under natural-long-day (NLD) conditions were used for qRT-PCR. ML1, newly emerging leaf; ML2, expanding leaf; ML3, fully expanded leaf; ML1, ML2, and ML3 collectively referred to as mature leaf (ML); YL, young leaf; ASA, around the shoot apex. Scale bar, 15 cm. (B) and (C) Expression analyses of RID1 and SID1 in young and mature leaves under NLD conditions during vegetative stage. (D) Rhythmic expression of SID1. The rice Ubiquitin (UBQ) gene served as the internal control. Values are shown as means ± SEMs of three independent experiments. The open and filled bars at the top represent the light and dark periods, respectively. (TIF) [file pgen.1006642.s005.tif]

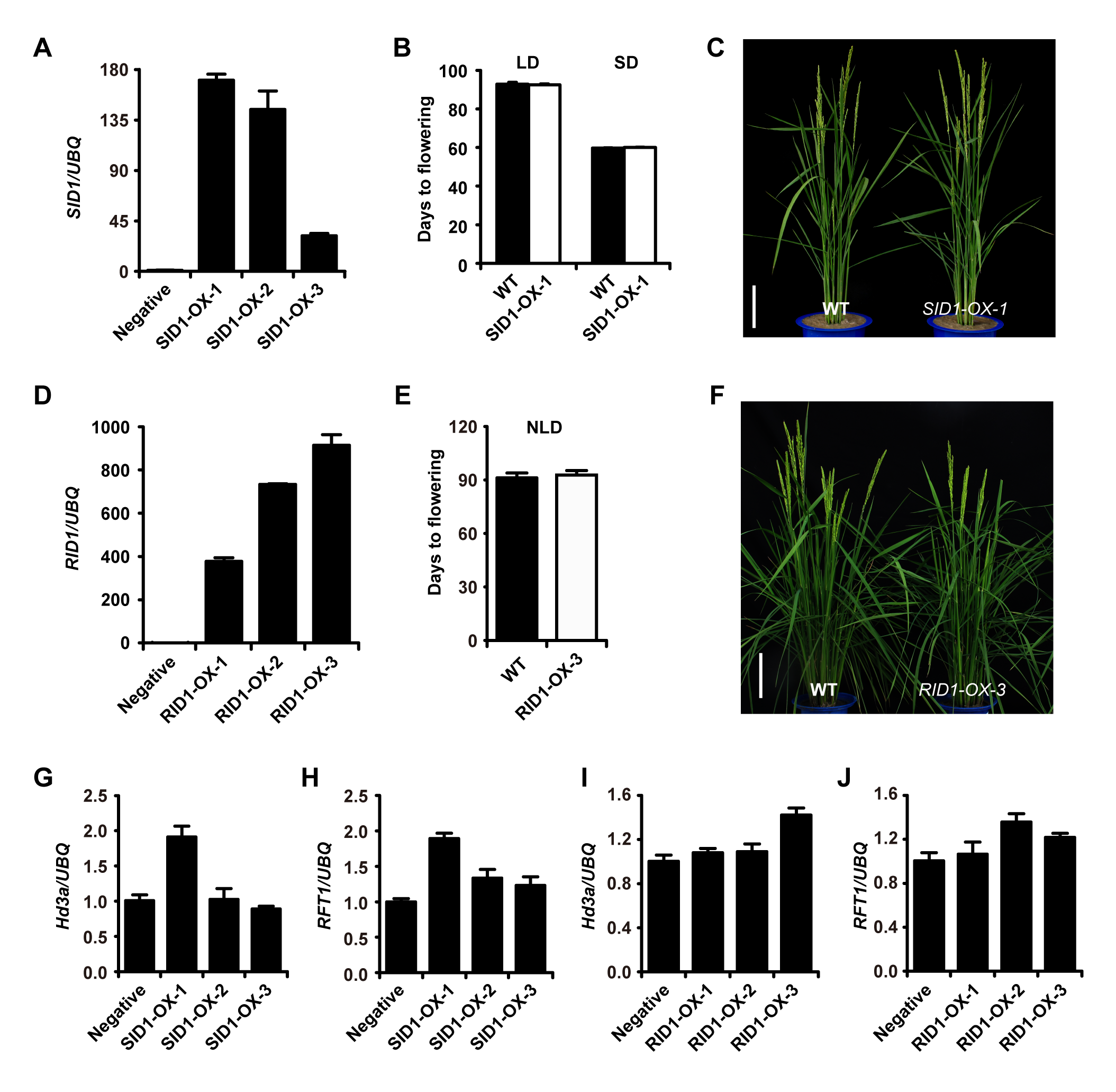

Supplement: S6 Fig — (A) Transcript analyses of SID1 in SID1-OX lines. Samples were harvested from 35-day-old plants under natural-long-day (NLD) conditions. Ubiquitin served as a control. Values are means ± SEMs of three replicate samples. (B) Days to flowering under long-day (LD, left) and short-day (SD, right) conditions. Black boxes, segregating wild type (WT); empty boxes, SID1-overexpressing lines (n = 10). (C) Phenotypes of segregating WT (left) and SID1-OX (right) plants at heading stage. Scale bar, 15 cm. (D) Transcript analyses of RID1 in RID1-OX lines. Samples were harvested from 35-day-old plants under NLD conditions. Ubiquitin served as a control. Values are means ± SEMs of three replicate samples. (E) Days to flowering under NLD conditions. Black boxes, segregating WT; empty boxes, SID1-overexpressing lines (n = 10). (F) Phenotypes of segregating WT (left) and RID1-OX (right) plants at heading stage. Scale bar, 15 cm. (G–J) Quantitative RT-PCR analysis of Hd3a and RFT1 in SID1 and RID1 overexpressing plants under NLD conditions. The transcript levels of each gene were normalized to the rice UBQ gene. Values are shown as means ± SEMs of three independent experiments. (TIF) [file pgen.1006642.s006.tif]

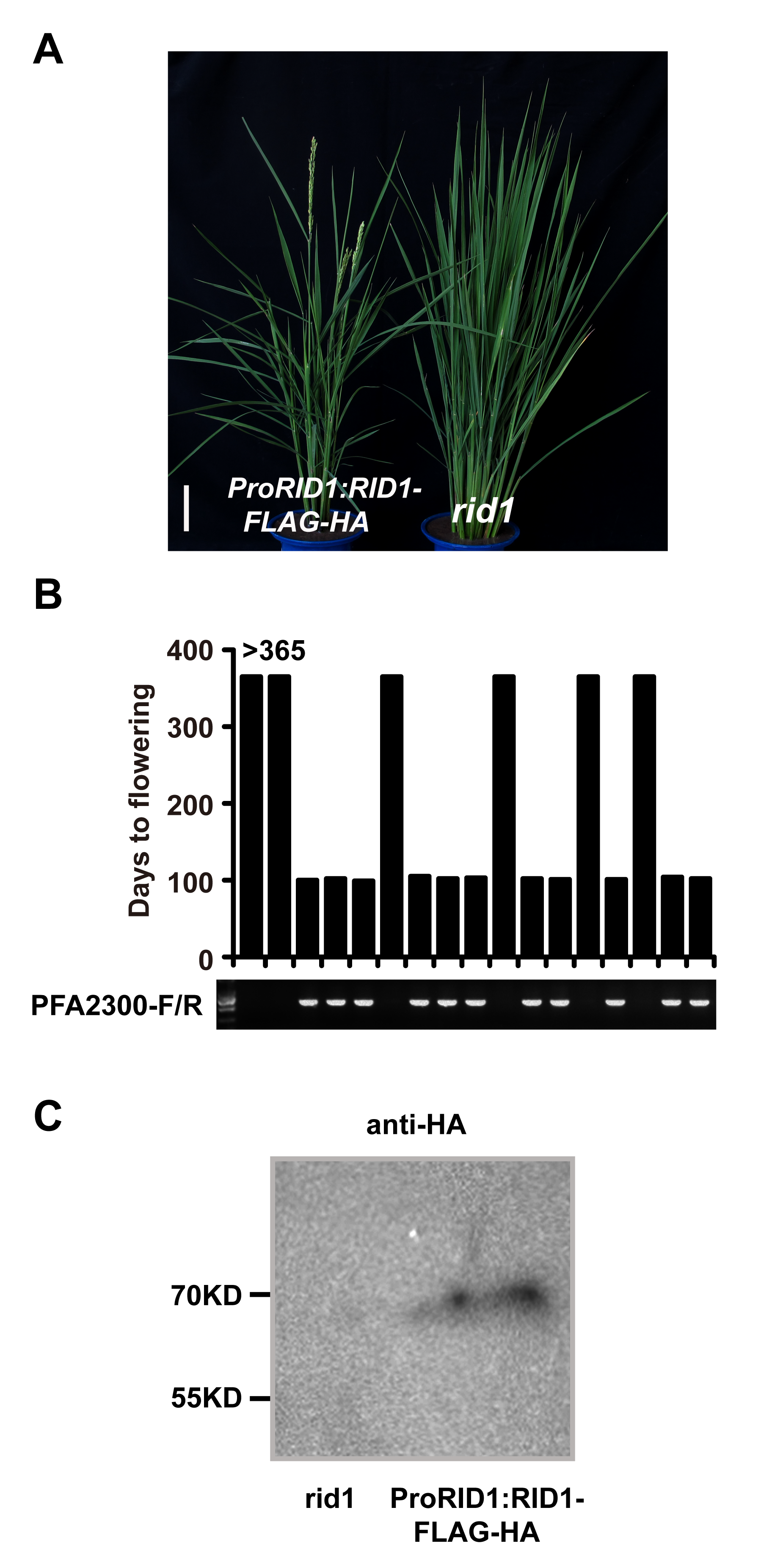

Supplement: S7 Fig — (A) Phenotypes of rid1 and ProRID1::RID1:FLAG:HA transgenic plants at heading stage. Scale bar, 15 cm. (B) Co-segregation between flowering time and the transgenic fragment in T1 segregants derived from a single copy restored line (T0). (C) Protein level of RID1 in rid1 and ProRID1::RID1:FLAG:HA transgenic plants. (TIF) [file pgen.1006642.s007.tif]

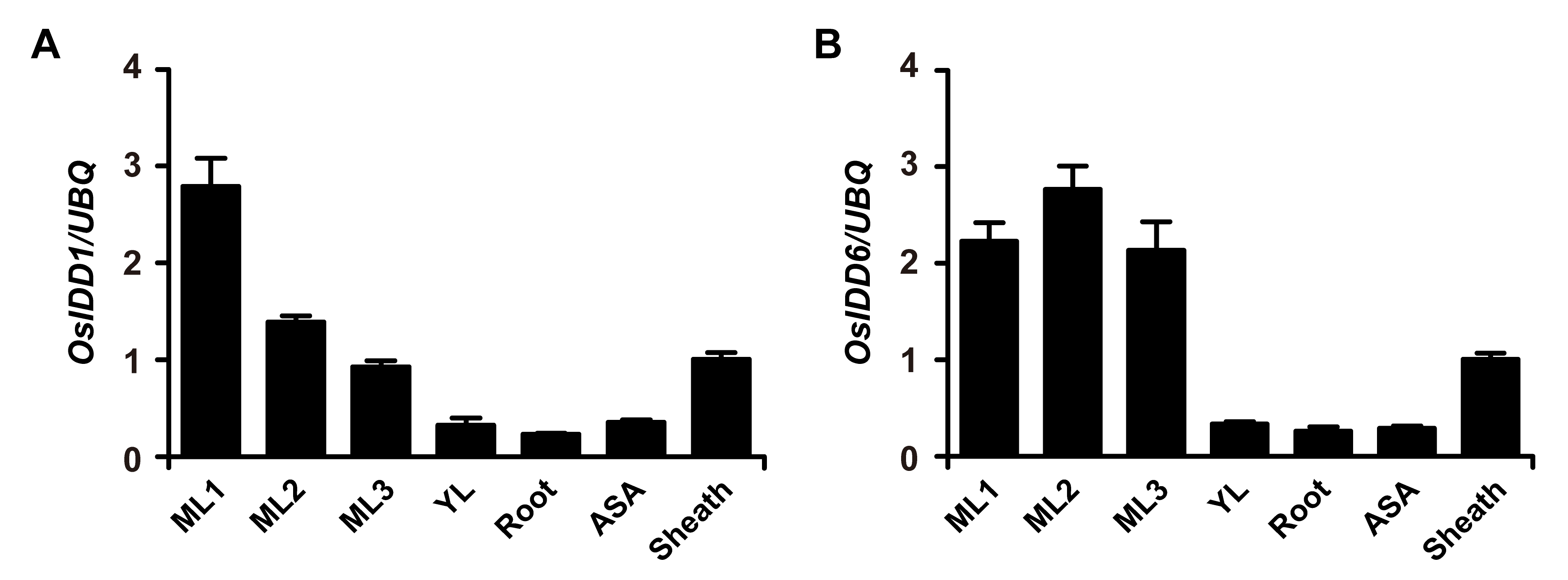

Supplement: S8 Fig — (A) Transcript levels of OsIDD1 in the indicated organs (S5A Fig). The data shown are the means ± SEMs of three independent experiments. (B) Transcript levels of OsIDD6 in the indicated organs. The data shown are the means ± SEMs of three independent experiments. (TIF) [file pgen.1006642.s008.tif]
