# Supplementary material for: Suppressor of rid1 (SID1) shares common targets with RID1 on florigen genes to initiate floral transition in rice
Source: PLoS Genet. 2017 Feb 24;13(2):e1006642. doi: 10.1371/journal.pgen.1006642 (PMC5345856; doi:10.1371/journal.pgen.1006642)
Supplement: S1 Table — (DOCX) [file pgen.1006642.s009.docx]

**S1 Table. Primers used in this study.**

| **Primer name** | **Sequence (5'–3')** | **Purpose** |
| --- | --- | --- |
| P4 | GGTGGGCCACTTCTAGCCGC | Genotyping |
| P5 | GTCCCCAGCTAGCGGCATGC |  |
| P6 | GCTGACCGCTTCCTCGTGCTTT |  |
| KAN-F | CGGCGATACCGTAAAGCAC |  |
| KAN-R | ACTGAAGCGGGAAGGGACT |  |
| M13-48 | GAGCGGATAACAATTTCACACAGG |  |
| CS | CAGGGTAAGCCGTTCCA |  |
| SID1-CE-F | TTCTTGCTTGAGTTTGTTATCG |  |
| SID1-CE-R | TGAAACTCGCTCCAACCG |  |
| PFA2300-F | CTGAAAACCCTAACCCTAATC |  |
| PFA2300-R | TATGGGTATCCCGCGTAATC |  |
| OsIDD4-OX-F | GGGGTACCGTAGAGTGTGGAAAGAAGGAAGCAAAAGGGGGAGA | Transgenic plants analysis |
| OsIDD4-OX-R | GGGGTACCGAGGGTTAACTGAATGACTGAAAGAGGACAAAAAATAGAAAA |  |
| OsIDD1-OX-F | TCCCCCGGGAAAGGCGTGACGCGAGCTTTTATGCT |  |
| OsIDD1-OX-R | CGGGATCCCAGTGGCAGCTACAGGACAAGAGCAAGG |  |
| OsIDD6-OX-F | CGGGATCCTGCAGGTCAGTCAGTTTGTCTGTCCTATGG |  |
| OsIDD6-OX-R | CGGGATCCGGCCTTAATTCGGTGTTATTTTTGATGGCT |  |
| Ehd1-OX-F | GGGGTACCCACAATGGGCTGCTCTAGTGAAGTGTTCG |  |
| Ehd1-OX-R | CGGGATCCCTAGAAATTCCAAAAACATGGTCCATATTCCTGTT |  |
| Hd3a-OX-F | GCTCTAGACTGCCTCTATCACAGTATATTTGCTCC |  |
| Hd3a-OX-R | GGGGTACCTTAGCCTTGCTCAGCTATTTAATTGC |  |
| PFA2300-RID1-F | GGGGTACCGGGAAAGCAAATGTTTAGCAGGAATGAGC |  |
| PFA2300-RID1-R | GGGGTACCGAAGTTGTGGCTCCACGTCTCGTTCAC |  |
| SID1(CDs)-OX-F | GGGGTACCCCATGGCATCCAACTCATCAGCG |  |
| SID1(CDs)-OX-R | CGGGATCCCGTCATTGCATCCTGCCTCCGT |  |
| RID1(cDNA)-OX-F | CGGGATCCCGATGGCGGCGGCGCAGGAGCCGAG |  |
| RID1(cDNA)-OX-R | GGGGTACCCCTTAGAAGTTGTGGCTCCACGTCTCG |  |
| SID1-GUS-F | CCCAAGCTTAGAACTCCATCCGGTCTCCTGTT |  |
| SID1-GUS-R | AACTGCAGAAATAGCGGCTTAATCTGGTCCTC |  |
| SID1-SgRNA-F | GTGTGCGTTGTTTGGAATTAGGGA |  |
| SID1-SgRNA-R | AAACTCCCTAATTCCAAACAACGC |  |
| SID1-SM-ZF1-F | CCGGTTCGTGGCCGAGGTGGCCAACAAGGG | Site-directed mutagenesis |
| SID1-SM-ZF1-R | CCCTTGTTGGCCACCTCGGCCACGAACCGG |  |
| SID1-SM-ZF2-F | TACCTGGCCCCGGAGCCGACGGCCGTCCAC |  |
| SID1-SM-ZF2-R | GTGGACGGCCGTCGGCTCCGGGGCCAGGTA |  |
| SID1-SM-ZF3-F | GAAGTGGAAGGCCGACAAGGCCTCCAAGCG |  |
| SID1-SM-ZF3-R | CGCTTGGAGGCCTTGTCGGCCTTCCACTTC |  |
| SID1-SM-ZF4-F | CGAGTACCGCGCCGACGCCGGCACCCTCTT |  |
| SID1-SM-ZF4-R | AAGAGGGTGCCGGCGTCGGCGCGGTACTCG |  |
| PM999-SID1-F | CGGAATTCATGGCATCCAACTCATCAGCGGCA | Subcellular  localization |
| PM999-SID1-R | GGGGTACCTTGCATCCTGCCTCCGTTGAAGGAC |  |
| SID1-LUC-F(B) | CGGGATCCATGGCATCCAACTCATCAGCG | DLR assay |
| SID1-LUC-R(E) | CGGAATTCTTGCATCCTGCCTCCGTTGA |  |
| SID1-LUC-N-R(E) | CGGAATTCGAGGCTGAGCGCCATGTTG |  |
| SID1-LUC-C-F(B) | CGGGATCCATGGCGCTCAGCCTCTCCC |  |
| None-SID1-F(B) | CGGGATCCATGGCATCCAACTCATCAGCG |  |
| None-SID1-R(E) | CGGAATTCTTGCATCCTGCCTCCGTTGA |  |
| Hd3a-LUC-F(H) | CCCAAGCTTAGCCCGTCTTGTTCAACCTTTCC |  |
| Hd3a-LUC-R(H) | CCCAAGCTTTACAATAGGTGGCAAATATGTTACATCACA |  |
| RFT1-LUC-F1 (H) | CCCAAGCTTATTCGGTGGGCGTAACTGGTA |  |
| RFT1-LUC-R1(B) | GAAGATCTAACTTTTGAAACTTCTCAAAATGCTT |  |
| 32a-RID1-F(B) | CGGGATCCATGTTGCTGTCTGATCTCTCGTCTGAT | EMSA |
| 32a-RID1-R(E) | CGGAATTCTTAGAAGTTGTGGCTCCACGTCTCGT |  |
| pGEX-4T-SID1-F(B) | CGGGATCC ATGGCATCCAACTCATCAGCGGCAGC |  |
| pGEX-4T-SID1-R(E) | CGGAATTC TCATTGCATCCTGCCTCCGTTGAAGGAC |  |
| Hd3a-EMSA-F | CTTATCTTGCCCGGACAAATCGAGCTAAGCAA |  |
| Hd3a-EMSA-R | TTGCTTAGCTCGATTTGTCCGGGCAAGATAAG |  |
| Hd3a-EMSA-MF | CTTATCTTGCCCGGATTAATCGAGCTAAGCAA |  |
| Hd3a-EMSA-MR | TTGCTTAGCTCGATTAATCCGGGCAAGATAAG |  |
| RFT1-EMSA-F | ATAACGTTTGACCATTTGTCTTATTAAATATATATATAAA |  |
| RFT1-EMSA-R | TTTATATATATATTTAATAAGACAAATGGTCAAACGTTAT |  |
| RFT1-EMSA-MF | ATAACGTTTGACCATTAATCTTATTAAATATATATATAAA |  |
| RFT1-EMSA-MR | TTTATATATATATTTAATAAGATTAATGGTCAAACGTTAT |  |
| ChIP-Hd3a-QRT-IF | ATGCCATTGATTTATTGACACAGC | ChIP-QPCR |
| ChIP-Hd3a-QRT-IR | TGATGCCTGCTTATTATGTAAATGTTC |  |
| ChIP-Hd3a-QRT-IIIF | TCCATCAAAACAACACACACCG |  |
| ChIP-Hd3a-QRT-IIIR | GCTCCCTCTTTTTCCAGATGTGA |  |
| ChIP-RFT1-QRT-IIF | GCATTTCAAAATGTACGACA |  |
| ChIP-RFT1-QRT-IIR | AATATGAAACAGAGAGAGTAAATGA |  |
| ChIP-RFT1-QRT-IVF | GGGGATTTGTCCTGATGTTTT |  |
| ChIP-RFT1-QRT-IVR | AAGCCTATTCACCCCCCTC |  |
| RID1-RT-F | TCGAGCTATTGTCGTCGTTG | RT-PCR |
| RID1-RT-R | GGAAGAGGGTGTACGTGTGC |  |
| Hd3a-RT-F | TCAGGGTTTTTTGCAAGATCGATGG |  |
| Hd3a-RT-R | ACGCTGCAGTAGTACCAGGAATATC |  |
| Ehd1-RT-F | CGAAAGCAAATGCAAGATCA |  |
| Ehd1-RT-R | TGGCAACTTGCTCTCTTGTC |  |
| GAPDH-F | CGACCCGTTCATCACCACCGAC |  |
| GAPDH-R | AGCTAGCAGCCCTTCCACCTCTCCA |  |
| Os02g45020-QRT-F | GAGGGACGCTCGTTTGAAGG | QRT-PCR |
| Os02g45020-QRT-R | TGTCGGCGAGCACCTTGTTA |  |
| Os02g45030-QRT-F | AAAGGCAGTGGCGAAGAGG |  |
| Os02g45030-QRT-R | CGTCCGTCCATCGACTTCC |  |
| Os02g45040-QRT-F | ACCAAGGCAGATGTGAGCG |  |
| Os02g45040-QRT-R | TTGAACAATGACTGAGGACGG |  |
| Os02g45054(OsIDD4)-QRT-F | ACAACCTCGGCTACATCGC |  |
| Os02g45054(OsIDD4)-QRT-R | ACTGGTCGGTGTTCGTCATC |  |
| Os02g45070-QRT-F | CGATGAGCGACTTGCATCTGT |  |
| Os02g45070-QRT-R | GGCCAATTCTTGGCAAGACAT |  |
| Os02g45080-QRT-F | AAAACGATATGCAGCCACAGCT |  |
| Os02g45080-QRT-R | CCCTACCTTGGCCCTTTTTATG |  |
| RID1-QRT-F | CGACGACAATAGCTCGATCGC |  |
| RID1-QRT-R | GTGCATGGTCACGGAGCCTT |  |
| Hd1-QRT-F | TCAGCAACAGCATATCTTTCTCATCA |  |
| Hd1-QRT-R | TCTGGAATTTGGCATATCTATCACC |  |
| Ehd1-QRT-F | GGATGCAAGGAAATCATGGA |  |
| Ehd1-QRT-R | AATCCCATCGGAAATCTTGG |  |
| Hd3a-QRT-F | CTTCAACACCAAGGACTTCGC |  |
| Hd3a-QRT-R | TAGTGAGCATGCAGCAGATCG |  |
| RFT1-QRT-F | TGACCTAGATTCAAAGTCTAATCCTT |  |
| RFT1-QRT-R | TGCCGGCCATGTCAAATTAATAAC |  |
| OsIDD1-QRT-F | CAGGGCAGCAGCAACAGCA |  |
| OsIDD1-QRT-R | CGGAGGACAGCATTGGGGA |  |
| OsIDD6-QRT-F | CCTTGTACAGCTCGTCCGACCA |  |
| OsIDD6-QRT-R | CGGCGGGCTTACCGTTCA |  |
| Ubiquitin-QRT-F | AACCAGCTGAGGCCCAAGA |  |
| Ubiquitin-QRT-R | ACGATTGATTTAACCAGTCCATGA |  |
